# Roles and Applications of Circular RNA in Virus Infection

**DOI:** 10.3390/ijms26199656

**Published:** 2025-10-03

**Authors:** Fang Gou, Yanmei Gao, Keke Zhong, Tian Bu, Yinggang Li, Faxiang Li, Rong Yang

**Affiliations:** 1 Engineering Research Center of Polyploid Fish Reproduction and Breeding of the State Education Ministry, College of Life Sciences, Hunan Normal University, Changsha 410081, China; 202470142961@hunnu.edu.cn (F.G.); 202320142793@hunnu.edu.cn (Y.G.); 202130081134@hunnu.edu.cn (K.Z.); butian@hunnu.edu.cn (T.B.); 202370142909@hunnu.edu.cn (Y.L.); 2 MOE Key Laboratory of Rare Pediatric Diseases, Center for Medical Genetics, School of Life Sciences, Central South University, Changsha 410081, China

**Keywords:** circular RNAs, viral infection, RNA therapy, viral circRNAs, host circRNAs

## Abstract

Circular RNAs (circRNAs) are novel types of covalently closed single-stranded RNA formed by the backsplicing of precursor mRNAs (pre-mRNAs). Recently, circRNAs have been shown to play a crucial role in various diseases, including cancers, cardiovascular and cerebrovascular diseases, and autoimmune diseases. Accumulating evidence has demonstrated that both host-derived and virus-encoded circRNAs play pivotal roles during viral infection, including modulating viral entry, genome replication, latency establishment, and the host antiviral immune responses while simultaneously facilitating viral immune evasion. However, their roles during viral infections and circRNA-host interactions remain to be further investigated. Therefore, this article reviews the key characteristics and biological functions of circRNAs, as well as recent advances in understanding the interactions between circRNAs from different sources and viral infections, which will offer insights for developing therapies targeting virus-associated diseases.

## 1. Introduction

Circular RNAs (circRNAs) are a class of endogenous noncoding RNAs that have long been overlooked but have gene expression regulatory functions. Although circRNAs were first discovered in plant-like viruses by Sanger et al. in 1976 [1], the fact that they are formed by backsplicing of precursor mRNAs—which is different from classical splicing—and the limitations of RNA sequencing technology at that time led to the common belief that circRNAs were simply byproducts of incorrect splicing [2]. It was not until 1993 that Capel and colleagues first identified a circular RNA transcript of the sex-determining gene Sry in the testes of adult mice [3]. With the development of genome sequencing technology and the advancement of RNA sequencing technology, our understanding of circRNAs has significantly improved [4,5,6]. The precise formation of circRNAs is governed by a backsplicing mechanism, which is facilitated by both cis-acting elements such as flanking complementary sequences and trans-acting factors including specific RNA-binding proteins [7,8,9]. CircRNAs can influence the biological activities of organisms through various mechanisms, and their expression levels are closely associated with developmental stages, physiological conditions, and a range of diseases. Their inherent stability in vivo, coupled with their species- and tissue-specific expression profiles, renders them exceptional diagnostic markers and therapeutic targets.

Research on viruses and circRNAs has emerged as a prominent topic within the field of noncoding RNAs, with a primary focus on the bidirectional regulatory roles of virus-encoded circRNAs and host-derived circRNAs during viral infections. On the one hand, virus-encoded circRNAs can influence disease progression by modulating various processes, such as viral replication, immune evasion, or latent infection. For example, Epstein–Barr virus (EBV)-encoded circRPMS1 enhances latent viral infection by sequestering host microRNAs through sponge mechanisms, whereas Kaposi’s sarcoma-associated herpesvirus (KSHV)-encoded circ-vIRF4 facilitates primary infection of the host. Similarly, studies have shown that several other viruses—including human papillomavirus (HPV), Merkel cell polyomavirus (MCPyV), hepatitis B virus (HBV), and Middle East respiratory syndrome coronavirus (MERS-CoV)—also utilize virus-encoded circRNAs to regulate aspects of their infectious processes. On the other hand, host-derived circRNAs play crucial roles in the antiviral immune response of the host. Numerous studies have revealed significant differences in the expression levels of specific circRNAs before and after viral infection, suggesting that these molecules may serve as novel biomarkers or therapeutic targets for managing viral infections.

Owing to their covalently closed circular structure, circRNAs are difficult to distinguish from linear transcripts via conventional RNA-seq. Low-abundance circRNAs are often masked by highly expressed linear RNAs, limiting detection sensitivity and accuracy. In terms of functional studies, circRNAs not only exhibit complex and diverse mechanisms of action but also demonstrate tissue-specific and time-specific expression levels, further complicating research efforts. Additionally, the precise mechanisms through which circRNAs operate in various diseases remain poorly understood, and their potential as biomarkers is impeded by issues related to sensitivity, specificity, and sample variability. These challenges collectively restrict the widespread application of circRNAs in clinical diagnosis and treatment.

Although technologies such as high-throughput sequencing and CRISPR screening have significantly advanced the discovery of virus-associated circRNAs, further research is essential to elucidate their generation mechanisms, validate their functions, and facilitate clinical translation. Future studies should concentrate on deciphering the molecular networks that underlie circRNA-virus interactions and exploring their potential applications in antiviral drug development. In this review, we summarize the primary characteristics and biological functions of circular RNAs, with a particular focus on the mechanisms of action of both virus-encoded and host-derived circRNAs during viral infections. We present a list of circRNA biomarkers and therapeutic targets for common viral infectious diseases while discussing the potential applications of circRNAs in vaccine development, gene editing, and cell therapy for treating viral infections. We aim to provide insights that will contribute to the development of more effective antiviral strategies.

## 2. The Main Characteristics of CircRNAs

**Figure 1:**
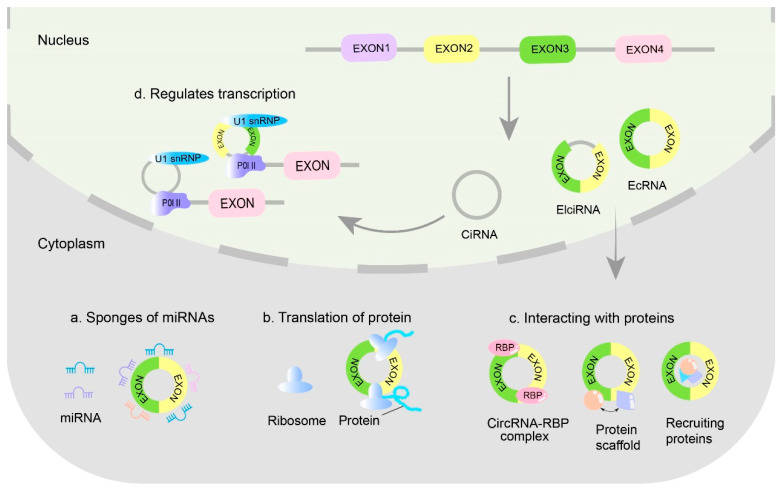
Classification and biological functions of circRNAs. EcRNA, exonic circRNA; EIciRNA, exon-intron circRNA; CiRNA, intronic circRNA. Intron-containing circRNAs function primarily within the nucleus to regulate transcription, enhancing the transcription of their parental genes and modulating alternative splicing. Exon-only circRNAs play diverse roles in the cytoplasm, including acting as miRNA sponges, interacting with proteins, and encoding polypeptides. (**a**) miRNAs act as microRNA sponges to modulate the posttranscriptional regulation of genes. (**b**) Encodes functional polypeptides that play roles in various cellular functions with the ribosome. (**c**) Interacts with proteins through several mechanisms: 1. CircRNAs act as decoys for RNA-binding proteins (RBPs), preventing their interaction with other RNA or DNA molecules by binding to them, thus regulating the expression of related genes. 2. They function as scaffolding molecules for proteins, aggregating different proteins to form complex protein complexes, which can influence protein interactions and functions. 3. Proteins are recruited to specific subcellular locations or organelles, thereby regulating protein function and localization. (**d**) By binding to RNA polymerase II, circRNAs potentially regulate the expression of parental genes at the transcriptional level. Solid Arrow: Represents formation and direction within the nucleus. Gradient Arrow: Represent transport from the nucleus to the cytoplasm and subsequent destinations.

CircRNAs are a type of noncoding RNA (ncRNA). They are covalently closed circular structures that lack a 5′ cap structure and a 3′ polyadenylate tail. When generated from pre-mRNAs through a back-splicing mechanism, they exhibit increased stability within the cellular environment. On the basis of their structural components and genomic origins, circRNAs can be categorized into three types: exonic circRNAs (EcRNAs), intronic circRNAs (ciRNAs), and exon-intron circRNAs (EIciRNAs). CircRNAs that contain introns primarily serve a transcriptional regulatory function within the nucleus, whereas circRNAs composed solely of exons fulfill various roles predominantly in the cytoplasm. We illustrate the classification and biological functions of circRNAs (Figure 1).

The most studied and abundant type of circRNA is EcRNAs, which account for approximately 80% or more of the total circRNA population and are enriched in the cytoplasm [10]. In contrast, a few ciRNAs and EIciRNAs are present in the nucleus. Owing to the absence of a polyA tail, circRNAs are resistant to degradation by nucleic acid exonucleases such as RNase R or other enzymes in vivo. This characteristic renders circRNAs significantly more stable than linear mRNAs, with a half-life that exceeds 48 h [11]. Researchers have reported that circRNAs are widely distributed and abundantly expressed. They are not only present in animal cells but also extensively distributed across fungi, plants (e.g., *Arabidopsis thaliana*), and protists [12]. In addition, circRNAs exhibit species- and tissue-specific expression patterns [13], and the expression patterns of some circRNAs are highly conserved among species.

## 3. The Main Biological Functions of CircRNAs

CircRNAs function as miRNA sponges, representing one of the key mechanisms underlying their gene regulatory roles. These circRNAs possess multiple miRNA binding sites distributed across their sequence, thereby competitively inhibiting miRNA-target gene interactions and precisely regulating the expression levels of downstream genes. For example, ciRS-7 harbors over 70 selectively conserved miRNA target sites, thereby modulating the expression of downstream target genes [14]. Research has demonstrated that circRNAs can undergo polypeptide translation through various mechanisms, including internal ribosome entry site (IRES)-initiated patterns, m6A internal ribosome entry site (MIRES)-initiated patterns, and UTR-mediated translation [15,16]. Yang’s group demonstrated that circRNA sequences in human cells are enriched with N6-methyladenosine (m6A) motifs and reported that a single m6A site is sufficient to drive circRNA translation [17]. Wang’s work revealed that inserting an IRES into the exonic fragment of a circRNA can drive the translation of circRNA-encoded proteins [18]. Previous studies have firmly established that circRNAs have significant potential for translation. For instance, researchers have recently identified circ-WRKY9 in rice plants infected with rice stripe mosaic virus (RSMV). This circRNA possesses two open reading frames (ORFs) and internal ribosome entry sites (IRES), enabling it to encode a polypeptide known as WRKY9-88aa, which consists of 88 amino acids [19]. In addition, certain circRNAs possess binding sites for specific enzymes or proteins, which enable them to interact with proteins in various ways and thereby participate in the regulation of essential biological processes such as cell growth and apoptosis. Qu’s work demonstrated through RIP experiments that circCDYL2 functions as a scaffold to bind the *EIF3D* protein, thereby regulating the initiation of *RAD51* translation [20].

## 4. Relationship of CircRNAs with Viral Infections

**Figure 2:**
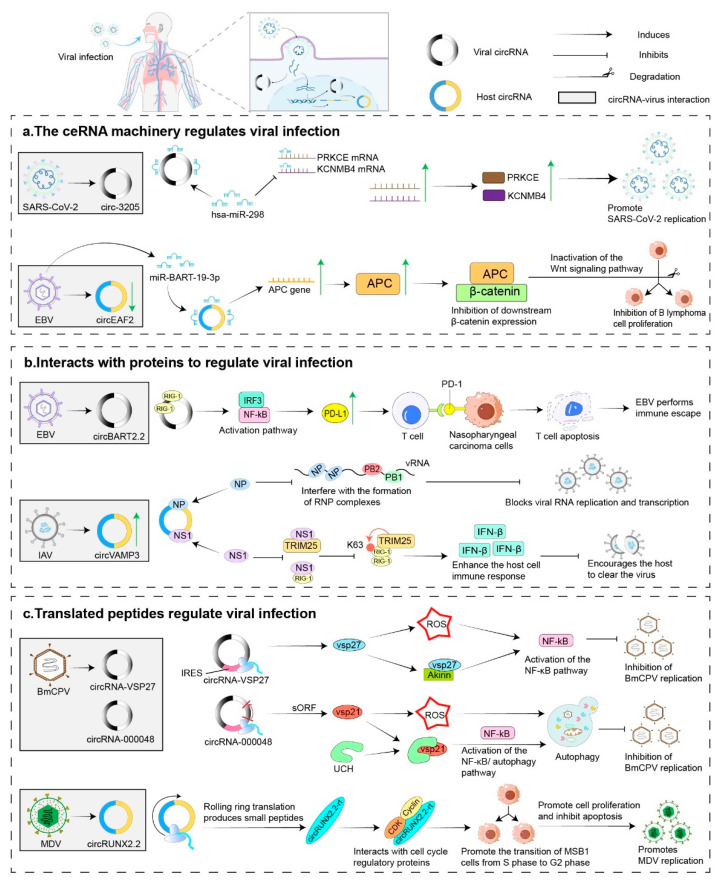
Roles of circRNAs in viral infections. (**a**) The ceRNA mechanism regulates viral infection: SARS-CoV-2-encoded circ_3205 sponges hsa-miR-298, promoting infection by upregulating PRKCE and KCNMB4 [21]. In EBV-positive B lymphoma cells, circEAF2 expression is significantly downregulated. CircEAF2 functions as a molecular sponge, effectively sequestering miR-BART19-3p. This interaction diminishes miR-BART19-3p’s ability to suppress *APC* mRNA, resulting in an upregulation of *APC* mRNA levels. The subsequent rise in APC protein levels leads to a decrease in β-catenin expression downstream, thereby suppressing B cell proliferation through Wnt pathway inactivation [22]. (**b**) Interacts with proteins to regulate viral infection: EBV-encoded circBART2.2 activates IRF3 and NF-kB by binding to RIG-I helicase domains, promoting PD-L1 expression for immune evasion [23]. In A549 cells infected with IAV, circVAMP3 levels increase significantly. It acts as a decoy for viral proteins NP and NS1, disrupting their functions. NP normally forms RNP complexes with viral RNA for replication and transcription. CircVAMP3 binds NP, preventing RNP formation and blocking viral replication. NS1 suppresses RIG-I activation by binding to TRIM25 or directly binding to RIG-1, thereby inhibiting the host immune response. CircVAMP3 binding to NS1 impairs its function, enhancing immune response and reducing viral RNA synthesis [24]. (**c**) Translated peptides regulate viral infection: CircRNA-vSP27 from BmCPV encodes vsp27, which induces ROS to activate NF-κB, inhibiting infection; vsp21, originating from vcircRNA_000048, induces cellular autophagy by activating ROS production to diminish viral replication [25]. MDV infection generates circRUNX2.2, which translates into peptide circRUNX2.2-rt to interact with cell cycle proteins, promoting cell proliferation and MDV replication [26]. PD-L1, programmed death ligand 1; PD-1, programmed death receptor 1; NP, nucleoprotein; NS1, nonstructural protein 1; RNP, ribonucleoprotein; ROS, reactive oxygen species; UCH, ubiquitin carboxy-terminal hydrolase; CDK, cyclin-dependent kinase; Cyclin, Cyclin-dependent kinase regulatory subunit. Green Arrow: Indicates increased expression of proteins or genes.

Viruses invade organisms via diverse mechanisms, proliferate, and replicate within host cells, thereby causing significant harm to the health of the organism. In recent years, with advancements in the study of circRNA structure and function, numerous scholars have reported that virus-derived circRNAs and differentially expressed host circRNAs are frequently detected in diseases associated with viral infections. These observations suggest that circRNAs may play critical roles in viral infection and host immune responses. Consequently, we provide a concise summary of the roles played by virus-encoded circRNAs and host gene-encoded circRNAs throughout the viral infection process (Figure 2).

### 4.1. Virus-Encoded CircRNAs and Their Roles in Viral Infection

Viruses are capable of generating virus-encoded circular RNAs (vcircRNAs) through host cell pathways. Zena Cai’s research team established the first database dedicated to viral circRNAs, known as VirusCircBase, which serves as a foundational map for exploring and studying viral circRNAs [27,28]. Owing to the complexity and diversity of circRNAs, as well as their technical challenges, the functional mechanisms of only a limited number of vcircRNAs have been validated through experimental studies. Therefore, this section provides a simple classification of vcircRNAs on the basis of existing studies and clarifies the potential roles they play in the viral infection process (Table 1).

#### 4.1.1. Plant Virus-Encoded VcircRNAs

In recent years, numerous circRNAs encoded by viral genomes have been discovered. However, to date, reports on circRNAs encoded by plant viruses are scarce, because the genetic material of plant viruses is predominantly single-stranded RNA (ssRNA). Additionally, circRNAs derived from RNA viruses may disrupt their viral genomes. Furthermore, plant viral genomes are relatively small, which may limit their capacity to encode circRNAs.

Viroids are pathogenic, single-stranded, covalently closed circular RNAs with complex secondary structures that do not encode proteins and depend on host factors for replication and symptom induction [55]. Potato spindle tuber viroid (PSTVd), identified and characterized in 1971, was the first discovered viroid [56]. Its nucleic acid includes a circular single-stranded RNA molecule that replicates within cells through a rolling circle mechanism. PSTVd infection hinders potato growth, resulting in morphological deformities in leaves and tubers, as well as a reduction in yield. Bao’s team conducted experiments demonstrating that siRNA derived from the virulence regulatory region (VMR) of PSTVd-RG1 can disrupt signalling pathways involved in gibberellin (GA) metabolism. This disruption leads to developmental retardation and results in the formation of small spindle-shaped tubers in plants [29].

#### 4.1.2. Animal Virus-Encoded VcircRNAs

Marek’s disease virus (MDV), a highly oncogenic herpesvirus, is capable of inducing fatal tumorous lesions in avian T lymphocytes. Through RNA sequencing technology, Alexis S. Chasseur and colleagues successfully identified various circular RNA molecules encoded by MDV. The expression hotspots of these circRNAs include the transcriptional units and latency-associated transcripts of the main viral oncogene encoding the Meq protein, which are closely associated with MDV virulence factors. These viral circRNAs may affect virus virulence and pathogenicity, although the specific mechanisms require further investigation [30].

*Bombyx mori* cytoplasmic polyhedrosis virus (BmCPV) is a typical double-stranded RNA virus with a single-layered capsid and is classified within the family Reoviridae, genus Orthoreovirus. CircRNA-vSP27 originates from the negative strand of the S5 segment dsRNA in the BmCPV genome. It inhibits viral multiplication by encoding the vSP27 peptide and activating the ROS/NF-κB signalling pathway. Additionally, the encoded vSP27 interacts with the nuclear protein Akirin, which further activates the NF-κB signalling pathway and delays viral multiplication [25]. VcircRNA_000048, derived from BmCPV infection, translates the vsp21 peptide in an IRES-dependent manner, and the peptide further delays viral replication via the NF-κB/autophagy pathway. Additionally, vcircRNA_000048 acts as a sponge for bmo-miR-2753, thereby regulating the expression of both host and viral genes [31].

*Bombyx mori* nucleopolyhedrovirus (BmNPV) belongs to the family Baculoviridae, genus Alphabaculovirus. Yaxin Zhang and colleagues confirmed that circRNA-000010, encoded by BmNPV, is translated into the VSP39 protein, which positively regulates viral replication [32].

Grass carp reovirus (GCRV) is also known as grass carp hemorrhagic virus (GCHV). In GCRV-infected Ctenopharyngodon idellus kidney (CIK) cells, GCRV encodes 32 circRNAs, which may function as sponges for miRNAs, thereby regulating host or viral genes to suppress viral replication [57]. Zeen Shen and colleagues discovered that circ_20, encoded by GCRV, interacts with BIP and PERK to form a circ_20-BIP-PERK ternary complex. This interaction enhances the binding between BIP and PERK, thereby inhibiting the PERK-eIF2α pathway and delaying GCRV replication and proliferation [33]. Recently, this team provided experimental evidence that circRNA-13, derived from the S8 genomic RNA of GCRV, plays a negative regulatory role in viral replication [34].

The cyprinid herpesvirus 2 (CyHV-2) causes goldfish hemorrhagic disease. Research has shown that the expression levels of the uracil DNA glycosylase (udg) gene of CyHV-2 are positively correlated with the proliferation of CyHV-2 and udg. Circ-udg encodes a truncated UDG protein, circ-udg-P147, which increases UDG protein levels to promote CyHV-2 replication [35].

White spot syndrome virus (WSSV) is a double-stranded DNA virus that primarily infects crustaceans and causes white spot disease in shrimp, characterized by extremely high mortality rates. Recent research has identified a translatable viral circRNA, circVP28, in WSSV-infected Litopenaeus vannamei. This circVP28 can be translated into the protein ceVP28, which can block the process of viral entry into the host cell [36].

#### 4.1.3. Human Virus-Encoded VcircRNAs

Nearly all cases of cervical cancer are associated with human papillomavirus (HPV) infection, which also causes other cancers, such as oropharyngeal, anal, and penile cancers. CircE7 is the only circular RNA encoded by HPV that has been identified to date. It undergoes m6A modification and is enriched in the cytoplasm [38]. Research has shown that circE7 is expressed in head and neck squamous cell carcinoma (HNSCC) and functions by downregulating the expression of the *LGALS9* gene, which encodes the galectin-9 protein. This downregulation suppresses cytotoxic T-cell activity, thereby promoting tumor immune evasion [37].

Epstein–Barr virus (EBV) infection can lead to the development of malignant epithelial tumors, including nasopharyngeal carcinoma and gastric cancer. The circBART2.2 gene, encoded by the EBV *BART* gene, binds to RIG-I to upregulate PD-L1 expression, promoting tumor immune evasion [23]. CircLMP2A acts as a sponge for miR-3908, regulating the expression of tripartite motif-containing protein 59 (TRIM59), thereby promoting cancer cell proliferation and metastasis [39]. CircLMP-2_e5, which is localized in both the cytoplasm and nucleus, is coexpressed with its homologous linear LMP-2 RNA during EBV lytic reactivation [40]. CircRPMS1 mediates the occurrence of nasopharyngeal carcinoma by sponging multiple miRNAs [41]. Additionally, CircRPMS1 recruits Sam68 to the METTL3 promoter, inducing the reverse activation of METTL3 and promoting the progression of EBV-associated gastric cancer [42]. Derived from the lytic *BHLF1* gene, circBHLF1 is located near the lytic replication origin (OriLyt). Its expression increases during reactivation and is associated with the OriLyt DNA sequence, suggesting a potential role in regulating lytic viral DNA replication [43].

Infection with Kaposi’s sarcoma-associated herpesvirus (KSHV) can lead to Kaposi’s sarcoma, primary effusion lymphoma (PEL), endothelial-to-mesenchymal transition-inducing tumors, and multicentric Castleman disease. Recently, circRNAs transcribed from the KSHV genome have been discovered, and their variety differs across virus life stages. Takanobu Tagawa and colleagues performed deep sequencing of RNA from the lymph nodes of KSHV-positive patients and revealed that circ-vIRF4 is highly expressed in these patients [44]. During the early stages of infection, circ-vIRF4 is packaged in antinuclease viral particles, predelivered, and plays a role during the initial phase of host invasion [45]. During the lytic replication phase of KSHV, circPANs are induced to be expressed. Their function is to bind to RNA-binding proteins, such as PABPC, causing these proteins to be relocated from the cytoplasm to the nucleus during viral infection, which is crucial for the effective late lytic expression of KSHV genes [46].

Approximately 80% of Merkel cell carcinoma (MCC) cases are associated with Merkel cell polyomavirus (MCV or MCPyV) infection. Over the past twenty years, the incidence of MCC has doubled, with the mortality rate reaching 30%. MCPyV encodes circALTO, which is localized in the cytoplasm and decorated with N6-methyladenosine (m6A). This circALTO gene encodes a variant of the ALTO protein, thereby promoting the transcription of host recombinant promoters and accelerating the MCPyV infection process [47]. Early MCPyV-infected cells are capable of encoding a circMCV-T sequence, as demonstrated by RNase R-resistant RNA sequencing. Although circMCV-T cannot be translationally expressed, it serves as a sponge for MCV-miR-M1, thereby facilitating the viral replication process [48,49].

Hepatitis B virus (HBV), a member of the Hepadnaviridae family of double-stranded DNA viruses, is a major cause of hepatitis, liver cirrhosis, and hepatocellular carcinoma. In 2018, Kazuma and colleagues reported that HBV generates circRNAs during replication. These circRNAs interact with the cellular DExH-box helicase 9 (DHX9). Knockout of DHX9 results in an increased abundance of HBV circRNAs, which subsequently leads to reduced levels of HBV surface proteins [58]. DHX9 clearly regulates HBV surface protein levels through HBV circRNA during HBV infection. In 2020, it was reported that HBV encodes a novel circular RNA, HBV_circ_1. HBV_circ_1 binds to cyclin-dependent kinase 1 (CDK1), thereby stimulating HBV proliferation, migration, and invasion. Consequently, patients positive for HBV_circ_1 present significantly higher mortality rates than do HBV_circ_1-negative patients [50,51].

Hepatitis C virus (HCV) infection initially leads to infectious viral hepatitis in humans. As the disease progresses, it can develop into severe end-stage liver diseases, including liver cirrhosis and liver cancer, which pose significant threats to human health. After HCV infects the human body, its genomic RNA is replicated and translated exclusively in the cytoplasm and does not integrate into the host chromosome. Recent studies have shown that the genomic RNA of cytoplasmic HCV is processed to produce virus-derived circular RNAs (vcircRNAs), which are categorized into three clusters and exhibit viral functions. The vcircRNAs containing an IRES (Cluster I) are translatable into proteins that aid the virus. Additionally, two high-abundance nontranslatable vcircRNAs (Clusters II and III) have been proven to increase viral RNA abundance. These findings suggest that the generated vcircRNAs regulate viral replication in HCV-infected cells [52].

Zena Cai and colleagues were the first to characterize the circRNA libraries encoded by three coronaviruses: Middle East respiratory syndrome coronavirus (MERS-CoV) and severe acute respiratory syndrome coronavirus 1/2 (SARS-CoV-1/2). They reported that MERS-CoV encodes significantly more circRNAs than SARS-CoV-1/2 does. Furthermore, the enrichment of circRNAs varies among different viruses depending on the stage of viral infection and various biological processes. This study suggested that different viral circRNAs may play distinct roles during the infection process [59]. A previous study demonstrated that circ_3205 is synthesized from the nucleocapsid (N) gene of SARS-CoV-2. Circ_3205 can act as a sponge for hsa-miR-298, increasing the mRNA levels of PRKCE and KCNMB4 to promote the progression of viral infection [21]. Virus-encoded circRNAs may contribute to dysfunction in host cells. For example, circSARS-CoV-2-N1368, a circRNA encoded by SARS-CoV-2, acts as a molecular sponge for miR-103a-3p, thereby activating the ATF7/TLR4/NF-κB signalling pathway. This activation increases the production of reactive oxygen species (ROS) in endothelial cells (ECs), ultimately resulting in oxidative damage and functional impairment of ECs in SARS-CoV-2-infected patients [53].

Influenza A virus H1N1 is an avian influenza virus subtype that can directly infect humans. The negative-sense RNA containing the nucleoprotein (NP) gene of H1N1 produces a circular RNA named circNP37. During viral infection, circNP37 acts as a sponge for host miR-361-5p, thereby positively regulating the expression of the viral *PB2* gene and promoting viral replication [54].

### 4.2. Role of Host CircRNAs in Viral Infections

#### 4.2.1. Viral Infections Alter Host CircRNA Expression Profiles

Viral infections alter host cell intracellular environments, affecting host circRNA expression profiles. Numerous studies have employed high-throughput sequencing and bioinformatics to analyze these changes across different viral infections, followed by experimental validation of the mechanisms through which these differentially expressed circRNAs function during infection. Following RSMV infection, circRNA-seq identified 56 differentially expressed circRNAs on chromosome 1 (22 upregulated, 34 downregulated). Functional studies highlighted circ-WRKY9, which is upregulated and encodes the WRKY9-88aa peptide that suppresses rice susceptibility to RSMV [19]. Similarly, in animal models, infection with Grass carp reovirus (GCRV) induced the differential expression of 76 circRNAs in CIK cells. Network analysis uncovered key circRNA-miRNA-mRNA axes that were significantly enriched in immune-related pathways, highlighting the critical role of these circRNAs in the host immune response to viral infection in fish [60]. Take bony fish infected with the *S. chuatsi rhabdovirus* as an example. In these fish, circSamd4a is significantly upregulated. Mechanistic insights reveal that circSamd4a functions as a competitive endogenous RNA by sequestering miR-29a-3p, which subsequently potentiates the STING-mediated NF-κB/IRF3 signaling pathway and strengthens the host’s antiviral immune response [61]. In human cells as well, a multitude of circRNAs have been found to exhibit significant changes in expression levels upon viral infection. HCMV infection of THP-1 cells results in the expression of 1421 differentially expressed circRNAs, which primarily regulate cell secretion, the cell cycle, and apoptosis. Among them, hsa_circ_0001445 and hsa_circ_0001206 exhibit significant differential expression in patients and may serve as potential biomarkers for HCMV infection [62]. In human umbilical vein endothelial cells infected with hantavirus, 70 differentially expressed circRNAs were identified. Among these, the circRNA-miRNA-mRNA axes such as circ_0002470-miR-3182-IFI44, circ_0006132-miR-1304-3p-OAS1, and circ_0000479-miR-337-3p-CASP1 were found to modulate viral infection by participating in the host cell’s innate immune response, type I interferon signaling pathway, and cytokine-mediated signaling pathway [63].

#### 4.2.2. Host CircRNAs in Viral Infection, Replication, and Pathogenicity

During viral infection, the expression levels of circRNAs in host cells undergo substantial alterations. These host circRNAs have the potential to play regulatory roles in various pathways associated with viral infection, replication, and pathogenesis.

**Figure 3:**
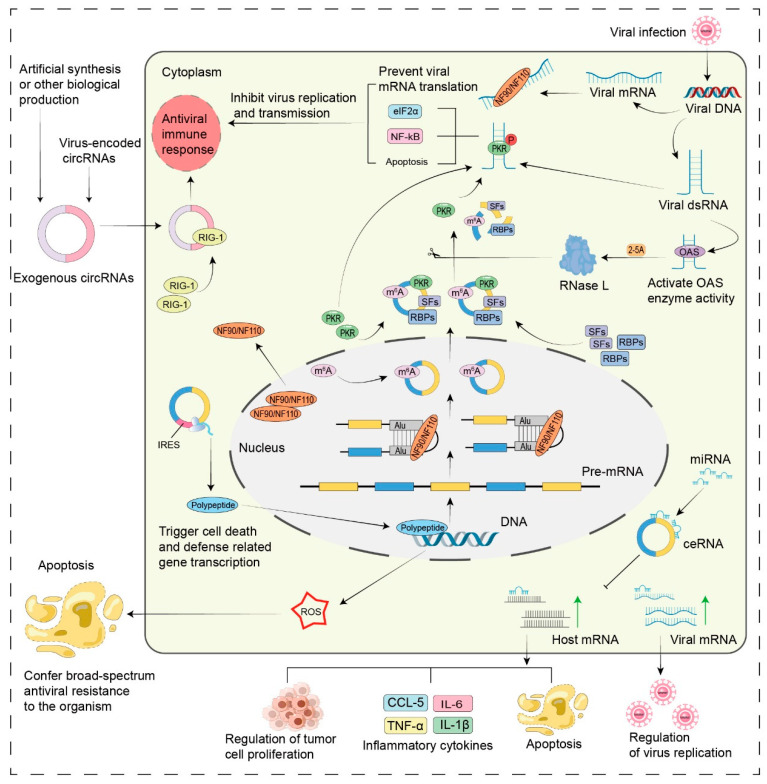
Roles of circRNAs in the host innate antiviral immune response. Under normal conditions, immune factors NF90 and NF110 within the nucleus of host cells stabilize intronic complementary sequences, facilitating the formation of circRNAs. These circRNAs undergo N6-methyladenosine (m6A) modification and are then transported to the cytoplasm, where they interact with proteins to inhibit RIG-I binding, preventing unnecessary immune activation. Upon viral infection, NF90/NF110 relocates from the nucleus to the cytoplasm, reducing circRNA synthesis and binding to viral mRNAs to obstruct replication [64]. Viral dsRNA activates 2′,5′-oligoadenylate synthetase (OAS), producing 2′-5′-oligoadenylate (2-5A) that stimulates RNase L activity for circRNA degradation. This process frees PKR to bind with viral dsRNA and initiate an antiviral response. PKR inhibits viral mRNA translation through phosphorylation and translational repression, phosphorylates eIF2α, activates NF-κB pathways, and induces apoptosis, thereby eliciting an innate antiviral immune response [66,69]. RIG-I can trigger an immune response by recognizing circRNAs that are not of host origin, which encompasses virus-derived circRNAs produced during infection as well as artificial circRNAs introduced experimentally [75,76]. CircRNAs also sequester microRNAs, influencing gene expression and modulating cellular behavior, including tumor cell proliferation and invasion. This interaction promotes the production of inflammatory cytokines, activates immune cells, and prevents virus dissemination by inducing cellular apoptosis. Peptides encoded by circRNAs can enter the nucleus to stimulate gene transcription related to cell death and defense, enhancing reactive oxygen species production. This cascade ultimately induces apoptosis and confers broad-spectrum antiviral resistance to the organism, such as circ-WRKY9 in rice, which possesses this function. m6A, N6-methyladenosine; SFs, Splicing Factors; RBPs, RNA-binding Proteins; RIG-I, Retinoic Acid-Inducible Gene I; dsRNA, Double-Stranded RNA; OAS, 2′-5′-Oligoadenylate Synthetase; 2-5A, 2′-5′-Oligoadenylate; RNase L, Ribonuclease L; PKR, Protein Kinase R; eIF2α, Eukaryotic Translation Initiation Factor 2 Alpha; NF-κB, Nuclear Factor Kappa-light-chain-enhancer of Activated B Cells; ROS, Reactive Oxygen Species. Black Gradient Arrow represents the formation process and destination. Green Arrow indicates increased mRNA expression. Black Solid Arrow with a Bar signifies inhibition. Black Scissor Arrow denotes degradation.

- Host circRNAs in regulating innate antiviral immune responses (Figure 3).

Intranuclear host circRNAs can function as molecular reservoirs for NF90/NF110. NF90/NF110 has been found to promote circRNA biogenesis by stabilizing complementary sequences of intronic RNAs in the nucleus. In response to viral infection, NF90 and NF110 are translocated from the nucleus to the cytoplasm, which results in a reduction in the production of circRNAs within the nucleus. Concurrently, NF90 and NF110 recognize and bind to viral mRNAs within the cytoplasm, thereby exerting their antiviral functions [64]. Host circRNAs released into the cytoplasm have been shown to act as inhibitors of double-stranded RNA (dsRNA)-activated protein kinase (PKR), a key regulator of innate immunity [65].

Upon viral infection, the presence of viral dsRNA triggers the activation of RNase L, resulting in the widespread degradation of intracellular circRNAs and the subsequent release of PKR [66]. The binding of viral dsRNA to the N-terminal binding domain of PKR has been shown to induce PKR dimerization and subsequent phosphorylation, leading to the activation of PKR [67]. Activated PKR can inhibit cellular mRNA translation by phosphorylating the eukaryotic initiation factor eIF2α, thereby suppressing viral protein synthesis [68,69]; it can also upregulate the expression of cytokines, including interferon, via the activation of the transcription factor NF-κB, leading to the widespread propagation of antiviral signals [70,71]; furthermore, it has the potential to induce apoptosis through intricate mechanisms, effectively blocking the viral transmission pathway [72,73]. Concomitantly, exogenous circRNAs have been demonstrated to upregulate the expression of multiple innate immune genes [74]. For example, the most typical immune receptor RIG-I recognizes exogenous circRNAs without m6A modification in the cell to trigger an immune response to suppress viral infection [75,76].

As a result, this enhanced innate immune response has been shown to increase cellular resistance to viral infections markedly. For example, research has demonstrated that circCBL in teleost fish upregulates the expression of the antiviral gene *MITA* by sequestering miR-125a-1-3p. This interaction subsequently activates the NF-κB and IRF3 signalling pathways, thereby increasing the production of antiviral genes and inflammatory cytokines. As a result, this mechanism strengthens the innate immune response of teleost fish to infections caused by Siniperca chuatsi rhabdovirus (SCRV) and Vibrio anguillarum [77]. However, the role of host circRNAs in antiviral immune responses is multifaceted, as they exhibit both stimulatory and inhibitory effects.

In some cases, host circRNAs have been shown to suppress immune responses or even facilitate pathogen replication. For example, Qiu and colleagues utilized IAV to infect the lung tissues of mice. In this model, circMerTK expression was significantly greater in the IAV-infected group than in the untreated control group. Further investigations revealed that circMerTK markedly suppressed the activation of type I interferon (IFN-β) and its downstream signalling pathways, consequently weakening the antiviral immune response of host cells and creating a more favorable environment for IAV replication [78]. Although the innate immune response of host cells serves as an effective defence mechanism against various types of viral invasion, viruses have evolved sophisticated strategies to evade host immune surveillance. For example, the EBV-encoded circBART2.2 facilitates immune escape by inducing apoptosis in tumor antigen-specific T cells, thereby compromising the host’s ability to mount an effective immune response against EBV [23].

- Host circRNAs as molecular sponges for virus-associated miRNAs.

In human umbilical vein endothelial cells infected with HTNV, circ_0000479 and circ_0046034 have been identified as ceRNAs that competitively bind to miR-149-5p. This interaction upregulates the expression of RIG-I, IL-6, and MXB, thereby inhibiting HTNV replication [63]. The expression of circEAF2 is significantly downregulated in EBV-positive B lymphoma cells. CircEAF2 acts as a competitive endogenous RNA (ceRNA) by sequestering microRNA-BART-19, thereby activating APC, inhibiting downstream β-catenin expression, and ultimately suppressing the Wnt signalling pathway to inhibit the proliferation of B lymphoma cells [22]. In HBV-infected patients, the expression level of circ_0004812 is significantly upregulated. This upregulation promoted the expression of Follistatin-like protein 1 (FSTL1) by competitively binding to miR-1287-5p, thereby attenuating interferon-induced immune responses [79].

Similarly, in HBV-positive hepatocellular carcinoma cells, circ-ATP5H increases TNFAIP3 levels by sequestering miR-138-5p, thereby promoting HBV replication and proliferation [80]. It is noteworthy that the ceRNA mechanism also plays a critical role in viral infections of non-human hosts. For instance, alphaherpesvirus pseudorabies virus (PRV) infection significantly upregulated the expression of circ29164, a circular RNA that does not encode a protein. Further investigation revealed that circ29164 competitively binds to ssc-miR-24-3p, thereby preventing it from targeting the mRNA encoding kelch-like ECH-associated protein 1 (KEAP1). This interaction maintains normal KEAP1 expression, which in turn induces intracellular caspase 3 activity and facilitates the release of cytochrome C from mitochondria. These events, which are key steps in the apoptosis pathway, ultimately lead to the inhibition of PRV replication [81].

- Host circRNAs: modulating viral infections through peptide translation and protein interactions.

A recent study revealed that circ-WRKY9, generated through backsplicing of the rice gene *OsWRKY9*, is significantly upregulated in rice plants infected with rice stripe mosaic virus (RSMV). circ-WRKY9 encodes a small 88-amino-acid peptide, WRKY9-88aa, which, upon entering the nucleus, activates the transcription of genes associated with cell death and defence mechanisms. This activation enhances reactive oxygen species (ROS) production, thereby contributing to the development of broad-spectrum disease resistance in rice plants [19]. Weiwei Zheng’s group discovered that circNLRP12 encodes a novel protein, NLRP12-119aa, in zebrafish infected with vesicular stomatitis virus (VSV). This protein interferes with the assembly of ribonucleoprotein (RNP) complexes, thereby inhibiting vesicular stomatitis virus (VSV) replication [82]. Jie Min and colleagues conducted a comprehensive analysis of circRNA transcription profiles in A549 cells infected with IAV and identified circVAMP3 as the most significantly upregulated circRNA. Subsequent investigations demonstrated that circVAMP3 directly suppresses viral replication by acting as a decoy for viral nucleoprotein (NP) and nonstructural protein 1 (NS1) [24]. In chickens with tumors induced by Marek’s disease virus (MDV), circRUNX2.2 is significantly upregulated in the spleen and is predominantly localized within the nucleus. This circular RNA recruits proteins that bind to the RUNX2 promoter, thereby enhancing RUNX2 transcription [83]. Furthermore, circRUNX2.2 can be translated into circRUNX2.2-rt, which interacts with proteins involved in cell cycle regulation, consequently promoting cell proliferation and inhibiting apoptosis [26]. These mechanisms may facilitate MDV replication and contribute to tumor development.

- Virus-induced changes in host circRNAs facilitate viral replication.

Upon infection of a host cell by a virus, the virus induces alterations in host circRNAs that remodel the intracellular microenvironment, thereby creating conditions conducive to viral replication. Researchers discovered that hsa_circ_0007321 was significantly downregulated in A549 cells infected with Zika virus (ZIKV). Subsequent investigations indicated that the downregulation of hsa_circ_0007321 diminished its sponge effect on miR-492, which resulted in the activation of the NF-κB signaling pathway and subsequently facilitated the replication of the Zika virus [84]. Zhang and colleagues subsequently conducted an in-depth investigation and demonstrated that MERS-CoV enhances the expression of circRNAs, including hsa_circ_0002846, hsa_circ_0002061, and hsa_circ_0004445, by exploiting the host cell protein heterogeneous nuclear ribonucleoprotein C (hnRNP C), thereby facilitating MERS-CoV replication [85]. Circ_0050463 can promote IAV replication by competitively binding to miR-33b-5p, thereby increasing the expression of eukaryotic elongation factor 1 alpha 1 (EEF1A1) [86]. According to a study by Liuyang Du et al., circTNFAIP3, which is derived from the *TNFAIP3* gene, is highly expressed in cells infected with deltacoronavirus. Subsequent experimental analyses demonstrated that circTNFAIP3 promotes deltacoronavirus replication through a specific mechanism involving the inhibition of apoptosis [87] (Table 2).

## 5. The Role of CircRNAs in the Diagnosis and Treatment of Viral Infections

### 5.1. CircRNAs as Diagnostic and Prognostic Tools for Viral Infection-Related Diseases

Viral infections can cause various diseases and, in severe cases, may even contribute to carcinogenesis. Common human oncogenic viruses include HPV, EBV, HBV, HCV, HHV-8, MCV, HTLV-1, and HCMV. Compared with linear RNAs, circRNAs are present in high concentrations in bodily fluids and tissues, and their structural characteristics increase their diagnostic potential for diseases [88]. Studies have revealed that the expression profiles of circRNAs in the host body undergo significant alterations following various viral infections. Many circRNAs found in blood or tissues have potential as biomarkers, aiding in the diagnosis and prognosis of viral infectious diseases [89,90,91,92,93]. By monitoring changes in these circRNAs, the viral replication status, host immune response, and efficacy of antiviral therapy can be evaluated, thereby providing a critical foundation for clinical decision-making. There is a list of circRNAs that are candidates for diagnostic biomarkers (Table 3).

Nasopharyngeal carcinoma (NPC), which is linked to Epstein–Barr virus (EBV), is difficult to detect and treat. Previous studies have shown that the expression level of hsa_circRNA_001387 is significantly increased in the peripheral blood, tumor tissues, and nasopharyngeal carcinoma cell lines of NPC patients and is closely correlated with the prognosis of NPC patients. Therefore, hsa_circRNA_001387 has potential as a biomarker for the diagnosis and prognosis of NPC [94]. Researchers have discovered that circCRIM1 is significantly upregulated in highly metastatic nasopharyngeal carcinoma (NPC) cells. Therefore, circCRIM1 is anticipated to serve as a potential biomarker and therapeutic target for predicting prognosis and overcoming treatment resistance in NPC patients [95]. EBV infection induces EBV-associated gastric cancer (EBVaGC). Previous studies have shown that hsa-circ0074362 is significantly downregulated in gastritis tissues and gastric cancer cell lines. Moreover, the expression level of hsa-circ0074362 is lower in gastric cancer patients than in gastritis patients [96]. These findings suggest that hsa-circ0074362 could serve as a novel diagnostic marker for gastric cancer. Other circular RNAs, such as hsa_circ_002059 [97], hsa_circ_0000520 [98], hsa_circ_0001017, and hsa_circ_0061276 [99], may serve as biomarkers for the diagnosis of gastric cancer and are involved in the development of gastric cancer.

Hsa_circ_0000976, hsa_circ_0007750, and hsa_circ_0139897 have been used to detect changes in plasma circRNA levels, and these three plasma circRNAs can serve as potential biomarkers for the diagnosis of HBV-associated hepatocellular carcinoma (HCC). Compared with alpha-fetoprotein (AFP), this method not only demonstrates superior accuracy in diagnosing small HCC but also identifies both small HCC and AFP-negative HCC cases [91]. The plasma levels of hsa_circ_0027089 can serve as a diagnostic marker for HBV-associated hepatocellular carcinoma (HCC) [100]. Circ-ATP5H facilitates HBV replication and expression through the regulation of the miR-138-5p/TNFAIP3 axis, making it a potential biomarker for both the diagnosis and treatment of HBV-related HCC [80]. Recent studies have identified a novel panel of circRNAs, including hsa_circ_0003288, circ-RNF13, circANRIL, circUHRF1, and hsa_circ_103047, as potential diagnostic biomarkers and therapeutic targets for HCV-associated hepatocellular carcinoma (HCC) [101].

HPV is a key predisposing factor for several cancers, including oral squamous cell carcinoma (OSCC) and cervical cancer (CC). In saliva samples from OSCC patients, the expression levels of hsa_circ_0001874 and hsa_circ_0001971 were significantly lower in postsurgery samples than in presurgery samples, suggesting their potential as diagnostic biomarkers for OSCC [102]. Cervical cancer (CC), caused by persistent infection with high-risk HPV, may also benefit from circRNA biomarkers; circYPEL2 [103] and hsa_circ_0065898 [104] have been identified as potential biomarkers for the clinical diagnosis and targeted therapy of CC.

The circRNA profiles in the peripheral blood of patients with community-acquired pneumonia (CAP), caused by a variety of pathogens, such as viruses, bacteria, and Mycoplasma, revealed that hsa_circ_0018429, hsa_circ_0026579, hsa_circ_0125357, and hsa_circ_0099188 were significantly upregulated. Notably, hsa_circ_0026579 can distinguish between viral pneumonia and nonviral pneumonia. These circRNAs may serve as effective biomarkers for CAP diagnosis and pathogen characterization [105].

Dengue fever, an acute insect-borne infectious disease caused by the Dengue virus (DENV), is characterized by distinct circRNA expression patterns in peripheral blood samples from patients. Specifically, the expression level of hsa_circ_0015962 was significantly greater after treatment than before treatment, whereas that of hsa_circ_0006459 was significantly greater before treatment than after treatment [106]. These findings indicate that the expression patterns of hsa_circ_0006459 and hsa_circ_0015962 are closely associated with the therapeutic response in dengue patients and may serve as potential biomarkers for both the diagnosis and evaluation of treatment efficacy in dengue fever patients.

Human adenovirus (HAdV)-associated severe pneumonia is highly lethal in children. Hsa_circ_0002171 is a potential diagnostic biomarker for HAdV pneumonia [107].

**Table 3:** CircRNAs as diagnostic and prognostic biomarkers for viral infections.

Virus	Diseases or Infections	Diagnostic and Prognostic Biomarkers	References
EBV	NPC	Hsa_circRNA_001387 is upregulated in EBV-infected NPC tissues.	[94]
		CircCRIM1 is significantly upregulated in highly metastatic NPC cells.	[95]
	GC	Hsa-circ0074362 is downregulated.	[96]
		Hsa_circ_002059 is downregulated.	[97]
		Hsa_circ_0000520 is downregulated.	[98]
		Hsa_circ_0001017 and hsa_circ_0061276 are downregulated.	[99]
HBV, HCV	HCC	Hsa_circ_0000976, hsa_circ_0007750, hsa_circ_0139897: For detecting plasma circRNA changes, potential biomarkers for HBV-HCC diagnosis.	[91]
		Hsa_circ_0027089 can discriminate HBV-related HCC from HBV-related cirrhosis and healthy participants.	[100]
		Circ-ATP5H is upregulated in HBV-infected HCC tissues.	[80]
		Hsa_circ_0003288, circ-RNF13, circANRIL, circUHRF1, hsa_circ_103047: Potential diagnostic biomarkers for HCV-HCC.	[101]
HPV	CC	CircYPEL2 and hsa_circ_0065898 are significantly upregulated.	[103,104]
	OSCC	Hsa_circ_0001874 and hsa_circ_0001971 are upregulated in the saliva of OSCC patients.	[102]
Various pathogens, including viruses	CAP	Hsa_cir_0018429, hsa_circ_0026579, hsa_cir_0125357, and hsa_circ_0099188 are upregulated. Hsa_circ_0026579 distinguishes viral pneumonia from nonviral pneumonia.	[105]
DENV	Dengue Fever	Hsa_circ_0006459 and hsa_circ_0015962 show significant changes before and after viral infection.	[106]
HAdVs	Pneumonia	Hsa_circ_0002171 can be used to diagnose highly pathogenic pneumonia.	[107]

Abbreviations: EBV, Epstein–Barr Virus; NPC, Nasopharyngeal Carcinoma; GC, Gastric Cancer; HBV, Hepatitis B virus; HCV, Hepatitis C virus; HCC, Hepatocellular Carcinoma; HPV, Human Papillomavirus; CC, cervical cancer; OSCC, Oral Squamous Cell Carcinoma; CAP, Community-Acquired Pneumonia; DENV, Dengue Virus; HAdVs, Human adenoviruses.

### 5.2. Antiviral Applications of CircRNA-Based RNA Therapies

#### 5.2.1. CircRNAs as Targets for Antiviral Therapy

**Figure 4:**
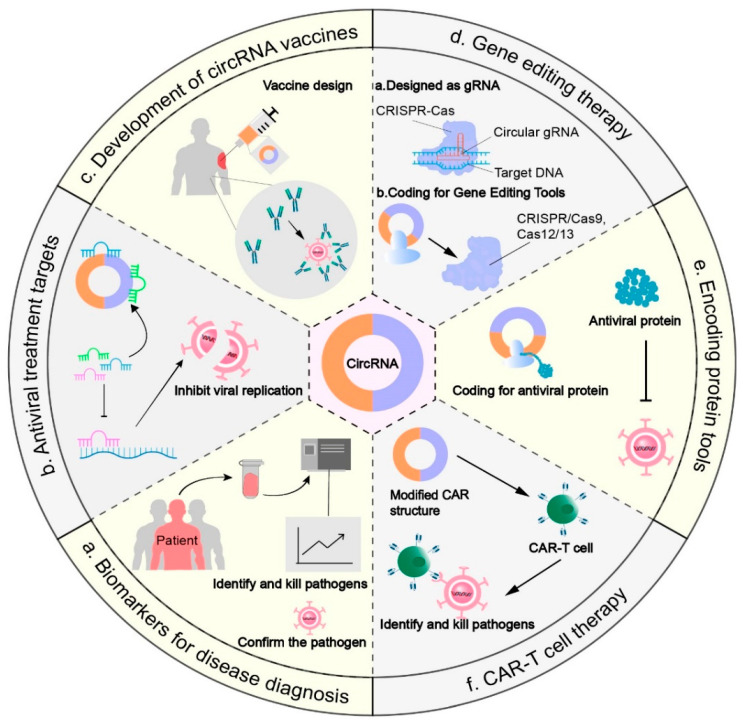
Antiviral applications of circRNA-based RNA therapies. (**a**) CircRNAs as biomarkers for disease diagnosis. (**b**) CircRNAs as antiviral treatment targets. (**c**) CircRNA vaccines. (**d**) CircRNAs in gene editing therapy. (**e**) CircRNAs as tools for protein encoding. (**f**) CircRNAs in CAR-T-cell therapy. Black Arrow represents the formation process and action pathway. Black Solid Arrow with a Bar indicates inhibition.

Given that circRNAs can participate in virus–host cell interactions by functioning as miRNA sponges, binding to viral genes or associated proteins, and so on, they have the potential to influence viral infection and pathogenicity (Figure 4). Thus, circRNAs can serve as therapeutic targets for viral infections, with several examples detailed (Table 4). In EBV-associated gastric cancer, circ-LMP2A attenuates the tumor-suppressive effect of the miR-3908/TRIM59/p53 axis [39]; circRPMS1 is highly expressed in gastric cancer tissues and may serve as a therapeutic target for EBVaGC [42]. Knockdown of circ-ATP5H inhibits HBV expression and replication in cancer cell lines from HBV-infected patients [80]; downregulation of circRNA_10156 [108] or circ-0015004 [109] significantly suppresses hepatocellular carcinoma cell growth. CircSORBS1 not only upregulates the level of RUFY3 protein, thereby inhibiting lung cancer progression [110], but also binds to miR-99, enhancing *GATA4* expression and subsequently promoting cardiomyocyte proliferation while reducing apoptosis [111]. Zhang’s investigation demonstrated that hsa_circ_0004812 sponges miR-1287-5p to upregulate *FSTL1* expression, thereby suppressing the host immune response in HBV-positive patients, suggesting its potential as a therapeutic target for chronic hepatitis B virus [79]. Hsa_circ_0001400 interacts with splicing factors to inhibit KSHV lytic transcription and replication [112,113].

Xi Zhang and colleagues reported that knockdown of circFNDC3B and circCNOT1 reduces MERS-CoV loads and suppresses ERK/MAPK/RIG-I-mediated antiviral signalling, indicating their potential as therapeutic targets against MERS-CoV infection [114]. Pfaffenrot et al. artificially synthesized an antisense RNA, the AS_1-75 circRNA, which targets and binds to the conserved sequence in the 5′-UTR of SARS-CoV-2 viral RNA. This interaction resulted in a 90% reduction in viral replication and proliferation, with the effect persisting for at least 48 h [115]. In hantavirus-infected endothelial cells, circ_0000479 inhibits viral replication by sponging miR-149-5p [63]. CircRNA-chr19 sponges Ebola virus miR-30b-3p to modulate *CLDN18* expression, enhancing viral recognition and suppression [116]. EV71 infection activates the hsa_circ_0069335/miR-29b/PMP22 axis to inhibit Schwann cell growth, suggesting that hsa_circ_0069335 is a potential therapeutic target for EV71-induced neuropathy [117].

Targeted drugs that inhibit or modulate circRNA expression could be designed on the basis of these principles, potentially blocking the viral infection process. For example, RNA interference and antisense oligonucleotides (ASOs) are employed to inhibit critical binding sites, whereas CRISPR-Cas13 is utilized for the knockdown of circular RNAs in the context of antiviral therapy [118,119]. Additionally, specific circRNAs could be artificially engineered to precisely target key regulatory nodes in the viral infection process, such as by serving as inhibitors of miRNA activity. The most typical example is that HCV replication depends on host miRNA-122. Oliver Rossbach’s group exploited this dependency by synthesizing an artificial circRNA to function as a miRNA-122 sponge, thereby significantly reducing the availability of miRNA-122 required for HCV replication and ultimately inhibiting both HCV replication and translation. This artificial circRNA was shown to reduce HCV titers in patients with an efficacy comparable to that of Miravirsen, the first anti-miRNA drug that reduces HCV titers by functionally sequestering miRNA-122 [120]. The high stability of these circRNAs in body fluids, their low immunogenicity, and the ease of efficient delivery via systems such as lipid nanoparticles (LNPs) enable more effective and safer antiviral therapies.

**Table 4:** CircRNAs as therapeutic targets for viral infections.

Virus	Diseases or Infections	Therapeutic Targets	References
EBV	GC	Inhibition of circ-LMP2A from EBV enhances tumor suppression.	[39]
		circRPMS1 as a potential therapeutic target in EBV-associated gastric cancer.	[41,42]
	NPC	CircCRIM1 sponges miR-422a to prevent its inhibition of the target gene *FOXQ1*, thereby promoting NPC metastasis and chemoresistance.	[95]
HBV, HCV	HCC	Circ-ATP5H sponges miR-138-5p to regulate TNFAIP3 expression, promoting HBV replication and expression; inhibiting circ-ATP5H from HBV slows liver cancer progression.	[80]
		Circ_0004812 is identified as a potential target for chronic hepatitis B infection.	[79]
		Circ-10156 acts as a molecular sponge for miR-149-3p, regulating the proliferation of HBV-related hepatocellular carcinoma cells via the miR-149-3p/Akt1 pathway. Inhibiting the expression of circ-10156 in hepatocellular carcinoma tissues suppresses cancer cell proliferation.	[108]
		Artificial circRNA sequesters miR-122, thereby inhibiting viral protein production in HCV cell culture systems. Relevant artificial circRNAs can also suppress HCV-related hepatocellular carcinoma.	[120]
		Knockdown of Circ-0015004 significantly inhibits hepatocellular carcinoma cell growth.	[109]
		CircSORBS1 inhibits lung cancer progression.	[110,111]
KSHV	KS	Hsa_circ_0001400 inhibits KSHV lytic transcription and replication.	[112,113]
MERS-CoV	LUAD	Knockdown of circFNDC3B and circCNOT1 reduces cellular viral load.	[114]
SARS-CoV-2	COVID-19	AS_1-75 circRNA targets the conserved 5′-UTR sequence of SARS-CoV-2 viral RNA, reducing viral replication by 90%.	[115]
HTNV	HFRS, HPS	Circ_0000479 sponges miR-149-5p to regulate RIG-I expression, inhibiting Hantavirus replication indirectly.	[63]
EBOV	EHF	CircRNA-chr19 targets and sequesters Ebola virus-associated miR-30b-3p, regulates *CLDN18* expression, and aids the immune system in recognizing and inhibiting viral replication.	[116]
EV71	HFMD	Hsa_circ_0069335 is a novel potential therapeutic target for EV71-induced neuronal diseases.	[117]

Abbreviations: KS, Kaposi Sarcoma; MERS-CoV, Middle East Respiratory Syndrome Coronavirus; LUAD, Lung Adenocarcinoma; COVID-19, Coronavirus Disease 2019; HTNV, Hantaan virus; HFRS, Hemorrhagic Fever with Renal Syndrome; HPS, Hantavirus Pulmonary Syndrome; EBOV, Ebola virus; EHF, Ebola Hemorrhagic Fever; EV71, Enterovirus 71; HFMD, Hand-foot-mouth disease.

#### 5.2.2. CircRNA Vaccines for Viral Infectious Diseases

The development of vaccines has progressed through several stages, beginning with traditional vaccine platforms such as inactivated and live attenuated vaccines. This evolution has led to the emergence of modern subunit vaccines, recombinant protein vaccines, and mRNA vaccines [121]. In recent years, circRNA vaccines have garnered significant attention as novel vaccine technologies because of their unique structural advantages and therapeutic potential. Compared with linear mRNA vaccines, circRNA vaccines exhibit not only enhanced stability and reduced immunogenicity [122] but also the ability to express antigens for an extended duration in vivo, thereby eliciting a prolonged immune response. Additionally, they possess advantages such as high thermal stability [123] and elevated translation efficiency [124]. These characteristics indicate significant potential for application in the treatment of viral infectious diseases.

CircRNA vaccines administered into the body can be translated into viral antigen proteins, thereby eliciting a robust immune response in the host and augmenting the body’s antiviral capabilities [123]. In 2022, the circRNA vaccine developed by Professor Wensheng Wei and his research team demonstrated strong protection against SARS-CoV-2 and its variants in both mice and rhesus monkeys [125]. Recently, Jinge Zhou and colleagues developed four monovalent circRNAs that encode monkeypox virus (MPXV) antigens: cirA29L, cirA35R, cirB6R, and cirM1R. These circRNAs can elicit comprehensive and effective protection against the monkeypox virus [126]. Jiawu Wan et al. developed a circRNA-G that targets lymph nodes and expresses the rabies virus glycoprotein (G). They also prepared mannose-lipid nanoparticles (mLNPs) specifically designed to target dendritic cells for effective delivery. These findings demonstrated that this mLNP-circRNA vaccine could elicit a robust immune response without compromising tissue targeting while simultaneously enhancing lyophilization stability and specificity [127].

CircRNA vaccines encoding multisubtype neuraminidase (NA) antigens from H1N1, H3N2, and influenza B viruses can induce broad-spectrum NA immunity against heterologous influenza strains, which is highly important for the development of broad-spectrum vaccines and the advancement of vaccine technology based on circRNAs [128]. Exogenous circRNAs not only function as platforms for antigenic expression but also enhance innate immune responses by integrating immunomodulatory molecules, including cytokines and pattern recognition receptors [74]. This dual functionality enables them to exert immune-enhancing effects akin to those of vaccine adjuvants [129]. For example, integrating the immunomodulatory molecules CXCL13 and influenza virus hemagglutinin (HA) into circRNAs for coexpression and delivering them to lymph nodes (LNs) via lipid nanoparticles can significantly increase the production of cross-reactive antibodies against both the influenza virus and SARS-CoV-2 [130].

When comparing circRNA vaccines to established mRNA platforms, considerations regarding production scalability and delivery are of paramount importance. In terms of manufacturing, while the upstream in vitro transcription processes are similar for both platforms, the downstream purification of circRNA—which necessitates the separation from linear RNA byproducts—currently presents greater challenges for large-scale production compared to mRNA purification. With respect to delivery, both platforms utilize lipid nanoparticles (LNPs); however, the inherent stability of circRNA may allow for lower dosing requirements. This characteristic has the potential to alleviate some dose-dependent delivery challenges that are typically associated with LNPs.

Although circRNA vaccines exhibit great potential in the treatment of viral infectious diseases, several challenges remain, including unclear in vivo degradation mechanisms, compromised stability and immunogenicity, and issues with low purity and inadequate targeting in delivery systems [131]. Moving forward, it will be necessary to refine circRNA technology, engineer novel delivery vectors, and explore combination therapeutic strategies involving adjuvants and multivalent antigens [132].

#### 5.2.3. CircRNAs for Gene Editing

Gene editing therapies treat genetic diseases by correcting pathogenic mutations. RNA-mediated short-acting editors can be transiently expressed, thereby minimizing off-target effects. Compared with conventional RNA molecules, circRNAs, which are more stable and have longer half-lives, can further enhance the therapeutic efficacy of gene editing therapies.

CircRNAs can serve as efficient platforms for encoding gene-editing tools. For example, Ronghong Liang’s group developed novel guide editing systems (CPEs) by linking multiple CRISPR RNAs and RTT-PBS sequences in a circular RNA expression framework. These systems have achieved precise guide editing in various cell lines [133]. As another example, Chan-I Su et al. demonstrated the anti-RNA virus potential of a circRNA encoding *erCas13*, which targets the endoplasmic reticulum (ER). Specifically, the *erCas13* protein, guided by sgRNA, localizes to the ER and inhibits flavivirus replication [134]. CircRNAs can also be engineered as circular guide RNAs (cgRNAs) for guiding CRISPR systems for gene editing. Because gRNAs in gene editing systems are easily degraded by nucleases, circular gRNAs have higher stability and editing efficiency than linear gRNAs [135,136].

For example, in 2022, Professor Wensheng Wei’s research team developed circular ADAR-recruiting guide RNAs (cadRNAs) that can effectively recruit endogenous ADAR enzymes through the formation of double-stranded structures. This innovative approach achieved efficient RNA editing in both in vitro and in vivo experiments, demonstrating a significantly higher editing efficiency than conventional linear guide RNAs [136,137]. Recent studies have engineered a cgRNA for *Cas12f* [138] or utilized circRNA to guide a reverse prime editor [139]. These advances continually verify the great potential of circRNAs in gene-editing therapies.

#### 5.2.4. CircRNA Translation Platforms for Viral Infection Therapy

Unlike the conventional mRNA translation mechanism, circRNA translation initiation does not rely on the 5′ cap structure. Instead, it is initiated through an internal ribosome entry site (IRES) or m^6^A modification [15,17,140,141,142]. Multiple studies have shown that circRNAs with infinite open reading frames (ORFs) can synthesize proteins via both prokaryotic [143] and eukaryotic [144] translation systems. Even chemically synthesized circRNAs with unnatural phosphoramidate linkages exhibit rolling circle translation [145]. Recent research has also indicated that the exon junction complex (EJC), which is formed after splicing, can mediate cap-independent translation of circRNAs [146]. Collectively, these studies strongly support the notion that circRNAs possess substantial translational potential.

The application of circRNAs as translation platforms in antiviral therapy is thus worth exploring. On the one hand, circRNAs can be translated into antiviral proteins in vivo, directly inhibiting viral replication or enhancing the host’s antiviral capabilities. The unique cap-independent translation mechanism of circRNAs allows them to maintain the normal translation of proteins under unfavorable conditions such as viral infection or cellular stress [147]. For example, in rice infected with RSMV, the small peptide WRKY9-88aa, which is translated from circWRKY9 in the host, induces broad-spectrum disease resistance in rice plants [19]. Similarly, in zebrafish infected with VSV, the NLRP12-119aa peptide encoded by circNLRP12 directly inhibits viral replication [82]. One study utilized circRNAs synthesized in vitro to express viperin, a known broad-spectrum antiviral protein, and reported that it effectively inhibited the replication of the JEV-GFP virus [134]. On the other hand, circRNAs can be utilized to express immune-activating factors such as interferons, thereby enhancing the body’s antiviral immune response. CircRNAs encoding IL-12 [148] are delivered to the tumor microenvironment to enable efficient IL-12 expression, which promotes the proliferation of cytotoxic T cells and activates antitumor immunity [149]. This approach, which is based on circRNA cytokine immunotherapy, also holds promise for potential applications in antiviral therapy.

#### 5.2.5. CircRNAs for CAR-T and TCR Engineering

Chimeric antigen receptor (CAR) T-cell therapy modifies a patient’s T cells to recognize and eliminate diseased cells specifically. CircRNAs not only enable large-scale production of base-edited CAR-T cells [150] but also modulate key pathways related to CAR-T-cell exhaustion, persistence, and specificity, thereby increasing the versatility and applicability of CAR-T-cell therapies [151,152]. Unlike CAR-T cells, which are restricted to recognizing proteins on the cell membrane surface, TCR-T cells can identify both intracellular and cell surface proteins, thereby offering a broader spectrum of target options. CircRNAs can not only effectively activate antigen-specific T cells but also substantially increase the screening efficiency and expression stability of TCRs. Lianghua Shen and colleagues transfected circRNAs encoding CMVpp65 into monocyte-derived dendritic cells (moDCs), efficiently screening for antigen-specific T cells and ultimately achieving the expression of exogenous TCRs. These circRNA-based cm-pp65-TCR-T cells demonstrated a more sustained antigen-specific response in vivo and effectively controlled viral infections [153].

## 6. Conclusions and Prospects

In recent years, significant advancements have elucidated the multifaceted roles of circRNAs in viral infections. As a class of noncoding RNAs with covalently closed circular structures, circRNAs have emerged as key players in viral pathogenesis owing to their ability to modulate gene transcription and posttranscriptional regulation. In this review, we systematically summarize the molecular signatures and biological functions of circRNAs, with a particular focus on the dual regulatory paradigms adopted by virus-encoded and host-originated circRNAs: while viral circRNAs facilitate immune evasion or enhance viral replication, host circRNAs counteract these infections through miRNA sequestration and the activation of signalling pathways. Furthermore, we have identified promising circRNA biomarkers and therapeutic targets associated with viral infections (e.g., HBV, HCV, and EBV). Finally, given the stability and regulatory properties of circRNAs, we explore their innovative applications in antiviral vaccine development, CRISPR-based gene editing, protein expression modulation, and engineered cell therapy—findings that provide valuable insights for designing novel antiviral strategies.

Although significant progress has been made in understanding circRNAs during viral infections, translating these discoveries into clinical applications faces persistent challenges. Key limitations include the incomplete elucidation of circRNA regulatory mechanisms within virus–host interaction networks, which obscures their precise therapeutic targeting; the heterogeneity of circRNA expression profiles across host species, reducing their reliability as universal diagnostic biomarkers; and unresolved technical barriers in circRNA-based therapies, particularly inefficient in vivo delivery systems and unpredictable molecular persistence.

To overcome these challenges, future research must achieve breakthroughs in four directions: (1) elucidate the spatiotemporal regulatory networks of circRNAs within virus–host interactions by integrating single-cell and spatial transcriptomics to reveal their cell type-dependent functions; (2) surpass the sensitivity and specificity limits of current detection technologies by developing rapid platforms based on nanopore sequencing and CRISPR-Cas systems for accurate quantification of low-abundance circRNAs in clinical samples; (3) optimize in vivo delivery of circRNAs through engineered exosome carriers or lipid nanoparticles to enhance tissue targeting while systematically evaluating safety concerns such as immunogenicity and off-target effects; and (4) explore innovative design strategies for circRNA vaccines, including multivalent antigen presentation systems and immunomodulators. By converging multiomics analyses, artificial intelligence models, and advanced delivery technologies, circRNAs have emerged as a new generation of core targets for antiviral diagnosis and therapy, offering innovative solutions for precision medicine.

## Figures and Tables

**Table 1:** Viral circular RNAs and their roles in viral infection.

Host Type	Virus	Specific Host or Human Tissue	circRNA	Validation Type	Role	References
Plant	PSTVd	Potato (*Solanum tuberosum*)	The genome contains a circular RNA molecule.	In vivo and Sequencing-based	Facilitates viral replication in a rolling-circle replication way within host cells	[29]
Animal	MDV	*Gallus gallus domesticus*	MDV-circRNAs	In vitro	Closely associated with MDV virulence factors; influences the virulence and pathogenicity of the virus.	[30]
	BmCPV	*Bombyx mori*	circRNA-vSP27	In vitro	Inhibits viral replication by activating the NF-κB signalling pathway.	[25]
			vcircRNA_000048	In vitro	VcircRNA_000048 translates a small peptide vsp21 in an IRES-dependent manner and acts as a miRNA sponge to delay viral replication.	[31]
	BmNPV		circRNA-000010	In vitro	Promotes viral replication by translating into VSP39.	[32]
	GCRV	*Ctenopharyngodon idellus*	circ_20	In vitro	Circ_20 forms a circ_20-BIP-PERK ternary complex to delay the replication and proliferation of GCRV.	[33]
			circRNA-13	In vitro	Inhibits viral replication.	[34]
	CyHV-2	*Carassius auratus*	circ-udg	In vitro	Circ-udg encodes the circ-udg-P147 peptide to elevate UDG protein levels, thereby promoting CyHV-2 replication.	[35]
	WSSV	*Litopenaeus vannamei*	circVP28	In vivo	CircVP28 encodes the protein ceVP28, which blocks the entry of viruses into host cells.	[36]
Human	HPV	Head and neck squamous epithelium	circE7	In vitro	CircE7 promotes immune evasion in head and neck squamous cell carcinoma.	[37,38]
	EBV, HHV-4	Nasopharyngeal epithelium	circBART2.2	In vitro	Promotes immune evasion in nasopharyngeal carcinoma by upregulating PD-L1 through interaction with RIG-I.	[23]
		Gastric epithelium	circ-LMP2a	In vitro	Circ-LMP2a induces the stemness of EBV-associated gastric cancer by acting as a sponge for hsa-miR-3908.	[39]
		B lymphocytes	circLMP-2_e5	Sequencing-based	CircLMP-2_e5 is coexpressed with linear LMP-2 RNA during EBV lytic replication and may play a role in the EBV life cycle, although its specific function requires further investigation.	[40]
		Nasopharyngeal epithelium and Gastric epithelium	circRPMS1	In vitro	CircRPMS1 induces the reverse activation of METTL3 to promote EBV-associated gastric cancer progression.	[41,42]
		B lymphocytes	circBHLF1	Sequencing-based	Regulates lytic virus DNA replication.	[43]
	KSHV	Endothelial tissue and B lymphocytes	circ-vIRF4	Sequencing-based	Facilitates viral invasion of the host.	[44,45]
		B lymphocytes	circPANs	In vitro	Facilitates effective lytic activity of KSHV genes in the late phase.	[46]
	MCV	Skin epithelium	circALTO	Sequencing-based	Encodes the ALTO protein variant, is negatively regulated by miRNAs, and participates in viral transcriptional activation and pathogenesis.	[47]
		Skin epithelium	circMCV-T	Sequencing-based	Acts as a sponge for MCV-miR-M1.	[48,49]
	HBV	Hepatocytes	HBV_circ_1	In vitro and Sequencing-based	HBV circRNA 1 interacts with CDK1 to regulate cell proliferation.	[50,51]
	HCV	Hepatocytes	cluster I circRNAs	Sequencing-based	VcircRNAs that contained the viral internal ribosome entry site were found to be translated into proteins that displayed proviral functions.	[52]
		Hepatocytes	cluster II, III circRNAs	Sequencing-based	Nontranslated vcircRNAs were shown to enhance viral RNA abundance.	[52]
	SARS-CoV-2	Respiratory epithelium	circ_3205	Sequencing-based	Functions as a sponge for hsa-miR-298, upregulating *PRKCE* and *KCNMB4* genes to promote viral infection.	[21]
		Vascular endothelium	circSARS-CV2-N1368	In vitro	CircSARS-CoV-2-N1368 activates the ATF7/TLR4/NF-κB signalling pathway by functioning as a molecular sponge for miR-103a-3p, thereby causing oxidative damage and dysfunction in endothelial cells (ECs).	[53]
	H1N1	Respiratory epithelium	circNP37	In vitro	Functions as a sponge for host miR-361-5p to positively regulate viral replication.	[54]

Abbreviations: PSTVd, Potato spindle tuber viroid; MDV, Marek’s disease virus; BmCPV, Bombyx mori cytoplasmic polyhedrosis virus; BmNPV, Bombyx mori Nucleopolyhedrovirus; GCRV, Grass Carp Reovirus; CyHV-2, Cyprinid herpesvirus 2; WSSV, White Spot Syndrome Virus; HPV, Human Papillomavirus; EBV, Epstein–Barr Virus; HHV-4, Human Herpesvirus 4; KSHV, Kaposi’s Sarcoma Associated Herpesvirus; MCV, Merkel Cell Polyomavirus; HBV, Hepatitis B virus; HCV, Hepatitis C virus; SARS-CoV-2, Severe Acute Respiratory Syndrome Coronavirus 2; H1N1, Influenza A virus subtype H1N1.

**Table 2:** Summary of Host circRNAs with Antiviral or Pro-viral Functions in Viral Infections.

Pro-Viral or Antiviral CircRNA	CircRNA	Virus	Mechanism of Action	References
Antiviral circRNA	circ-WRKY9	RSMV (rice stripe mosaic virus)	Encodes peptide WRKY9-88aa to suppress rice susceptibility to RSMV.	[19]
	circSamd4a	S. chuatsi rhabdovirus (SCRV)	Acts as a ceRNA to sequester miR-29a-3p, enhancing STING-mediated NF-κB/IRF3 pathway for a stronger antiviral response.	[61]
	circ_0000479, circ_0046034479	HTNV (Hantaan virus)	ceRNAs bind to miR-149-5p, upregulate RIG-I, IL-6, MXB, inhibit HTNV replication	[63]
	circEAF2	EBV (Epstein–Barr virus)	ceRNA sequesters miR-BART-19, activates APC, inhibits β-catenin, suppresses the Wnt pathway, and inhibits B lymphoma proliferation	[22]
	circ29164	PRV (Pseudorabies virus)	Competitively binds to ssc-miR-24-3p, maintaining KEAP1 expression to induce caspase 3 activity and cytochrome C release, inhibiting PRV replication via apoptosis.	[81]
	circNLRP12	VSV (Vesicular stomatitis virus)	NLRP12-119aa inhibits VSV replication by disrupting RNP complexes.	[82]
	circVAMP3	IAV (Influenza A virus)	Directly suppresses viral replication by acting as a decoy for viral nucleoprotein (NP) and nonstructural protein 1 (NS1).	[24]
	circCBL	SCRV (Siniperca chuatsi rhabdovirus)	Sequestering miR-125a-1-3p upregulates *MITA* expression, activating NF-κB and IRF3 pathways to enhance teleost fish’s innate immune response.	[77]
Pro-viral circRNA	circMerTK	IAV (Influenza A virus)	IAV suppresses IFN-β activation and downstream signaling, weakening antiviral immunity and aiding its replication.	[78]
	circBART2.2	EBV (Epstein–Barr virus)	Induces apoptosis in tumor antigen-specific T cells to facilitate immune escape and compromise immune response against EBV.	[23]
	circ_0004812	HBV (Hepatitis B virus)	Upregulation promotes *FSTL1* expression by binding to miR-1287-5p, reducing interferon-induced immune responses.	[79]
	circ-ATP5H	HBV (Hepatitis B virus)	Increases TNFAIP3 levels by sequestering miR-138-5p, thereby promoting HBV replication and proliferation.	[80]
	circRUNX2.2	MDV (Marek’s disease virus)	circRUNX2.2-rt promotes cell proliferation and inhibits apoptosis by interacting with cell cycle proteins, facilitating MDV replication and tumor development.	[83]
	hsa_circ_0007321	Zika virus (ZIKV)	Downregulation of hsa_circ_0007321 activates the NF-κB pathway, promoting Zika virus replication.	[84]
	hsa_circ_0002846, hsa_circ_0002061, hsa_circ_0004445	MERS-CoV (Middle East respiratory syndrome coronavirus)	MERS-CoV uses hnRNP C to boost circRNA expression and promote its replication.	[85]
	circ_0050463	IAV (Influenza A virus)	It can promote IAV replication by competitively binding to miR-33b-5p, thereby increasing the expression of eukaryotic elongation factor 1 alpha 1 (EEF1A1).	[86]
	circTNFAIP3	Deltacoronavirus	Promotes deltacoronavirus replication through a specific mechanism involving the inhibition of apoptosis.	[87]

## Data Availability

Not applicable.

## References

[1] Sanger H.L., Klotz G., Riesner D., Gross H.J., Kleinschmidt A.K. (1976). Viroids are single-stranded covalently closed circular RNA molecules existing as highly base-paired rod-like structures. Proc. Natl. Acad. Sci. USA.

[2] Cocquerelle C., Mascrez B., Hétuin D., Bailleul B. (1993). Mis-splicing yields circular RNA molecules. FASEB J..

[3] Capel B., Swain A., Nicolis S., Hacker A., Walter M., Koopman P., Goodfellow P., Lovell-Badge R. (1993). Circular transcripts of the testis-determining gene Sry in adult mouse testis. Cell.

[4] Salzman J., Gawad C., Wang P.L., Lacayo N., Brown P.O. (2012). Circular RNAs are the predominant transcript isoform from hundreds of human genes in diverse cell types. PLoS ONE.

[5] Memczak S., Jens M., Elefsinioti A., Torti F., Krueger J., Rybak A., Maier L., Mackowiak S.D., Gregersen L.H., Munschauer M. (2013). Circular RNAs are a large class of animal RNAs with regulatory potency. Nature.

[6] Jeck W.R., Sorrentino J.A., Wang K., Slevin M.K., Burd C.E., Liu J., Marzluff W.F., Sharpless N.E. (2013). Circular RNAs are abundant, conserved, and associated with ALU repeats. Rna.

[7] Wasinska-Kalwa M., Mamot A., Czubak K., Frankowska K., Rajkiewicz A.A., Spiewla T., Warminski M., Pilch Z., Szulc-Gasiorowska M., Siekan K. (2025). Chemical circularization of in vitro transcribed RNA for exploring circular mRNA design. Nat. Commun..

[8] Gao X., Chen K., Wang H. (2025). NicOPURE: Nickless RNA circularization and one-step purification with engineered group II introns and cyclizing UTRs. Nucleic Acids Res..

[9] Belter A., Popenda M., Sajek M., Wozniak T., Naskret-Barciszewska M.Z., Szachniuk M., Jurga S., Barciszewski J. (2022). A new molecular mechanism of RNA circularization and the microRNA sponge formation. J. Biomol. Struct. Dyn..

[10] Patop I.L., Wüst S., Kadener S. (2019). Past, present, and future of circRNAs. EMBO J..

[11] Suzuki H., Zuo Y.H., Wang J.H., Zhang M.Q., Malhotra A., Mayeda A. (2006). Characterization of RNase R-digested cellular RNA source that consists of lariat and circular RNAs from pre-mRNA splicing. Nucleic Acids Res..

[12] Wang P.L., Bao Y., Yee M.C., Barrett S.P., Hogan G.J., Olsen M.N., Dinneny J.R., Brown P.O., Salzman J. (2014). Circular RNA Is Expressed across the Eukaryotic Tree of Life. PLoS ONE.

[13] Maass P.G., Glazar P., Memczak S., Dittmar G., Hollfinger I., Schreyer L., Sauer A.V., Toka O., Aiuti A., Luft F.C. (2017). A map of human circular RNAs in clinically relevant tissues. J. Mol. Med..

[14] Hansen T.B., Jensen T.I., Clausen B.H., Bramsen J.B., Finsen B., Damgaard C.K., Kjems J. (2013). Natural RNA circles function as efficient microRNA sponges. Nature.

[15] Fan X., Yang Y., Chen C., Wang Z. (2022). Pervasive translation of circular RNAs driven by short IRES-like elements. Nat. Commun..

[16] Wang Y., Wu C., Du Y., Li Z., Li M., Hou P., Shen Z., Chu S., Zheng J., Bai J. (2022). Expanding uncapped translation and emerging function of circular RNA in carcinomas and noncarcinomas. Mol. Cancer.

[17] Yang Y., Fan X.J., Mao M.W., Song X.W., Wu P., Zhang Y., Jin Y.F., Yang Y., Chen L.L., Wang Y. (2017). Extensive translation of circular RNAs driven by N^6^-methyladenosine. Cell Res..

[18] Wang Y., Wang Z.F. (2015). Efficient backsplicing produces translatable circular mRNAs. Rna.

[19] Pan X., Xu S.P., Cao G.H., Chen S.P., Zhang T., Yang B.B., Zhou G.H., Yang X. (2025). A novel peptide encoded by a rice circular RNA confers broad-spectrum disease resistance in rice plants. New Phytol..

[20] Qu H.K., Wang Y.M., Yan Q.J., Fan C.M., Zhang X.Y., Wang D., Guo C., Chen P., Shi L., Liao Q.J. (2024). CircCDYL2 bolsters radiotherapy resistance in nasopharyngeal carcinoma by promoting RAD51 translation initiation for enhanced homologous recombination repair. J. Exp. Clin. Cancer Res..

[21] Barbagallo D., Palermo C.I., Barbagallo C., Battaglia R., Caponnetto A., Spina V., Ragusa M., Di Pietro C., Scalia G., Purrello M. (2022). Competing endogenous RNA network mediated by circ_3205 in SARS-CoV-2 infected cells. Cell Mol. Life Sci..

[22] Zhao C.X., Yan Z.X., Wen J.J., Fu D., Xu P.P., Wang L., Cheng S., Hu J.D., Zhao W.L. (2021). CircEAF2 counteracts Epstein-Barr virus-positive diffuse large B-cell lymphoma progression via miR-BART19-3p/APC/β-catenin axis. Mol. Cancer.

[23] Ge J.S., Wang J., Xiong F., Jiang X.J., Zhu K.J., Wang Y.A., Mo Y.Z., Gong Z.J., Zhang S.S., He Y. (2021). Epstein-Barr Virus-Encoded Circular RNA CircBART2.2 Promotes Immune Escape of Nasopharyngeal Carcinoma by Regulating PD-L1. Cancer Res..

[24] Min J., Li Y., Li X., Wang M., Li H., Bi Y., Xu P., Liu W., Ye X., Li J. (2023). The circRNA circVAMP3 restricts influenza A virus replication by interfering with NP and NS1 proteins. PLoS Pathog..

[25] Zhang Y., Zhang X., Dai K., Zhu M., Liang Z., Pan J., Zhang Z., Xue R., Cao G., Hu X. (2022). Bombyx mori Akirin hijacks a viral peptide vSP27 encoded by BmCPV circRNA and activates the ROS-NF-κB pathway against viral infection. Int. J. Biol. Macromol..

[26] Wang L.L., Zheng G., Yang Y.Q., Wu J.F., Du Y.S., Chen J.H., Liu C.J., Liu Y.Z., Zhang B., Zhang H. (2024). Rolling-Translated circRUNX2.2 Promotes Lymphoma Cell Proliferation and Cycle Transition in Marek’s Disease Model. Int. J. Mol. Sci..

[27] Cai Z., Fan Y., Zhang Z., Lu C., Zhu Z., Jiang T., Shan T., Peng Y. (2021). VirusCircBase: A database of virus circular RNAs. Brief. Bioinform..

[28] Fu P., Cai Z., Zhang Z., Meng X., Peng Y. (2023). An updated database of virus circular RNAs provides new insights into the biogenesis mechanism of the molecule. Emerg. Microbes Infect..

[29] Bao S., Owens R.A., Sun Q., Song H., Liu Y., Eamens A.L., Feng H., Tian H., Wang M.B., Zhang R. (2019). Silencing of transcription factor encoding gene StTCP23 by small RNAs derived from the virulence modulating region of potato spindle tuber viroid is associated with symptom development in potato. PLoS Pathog..

[30] Chasseur A.S., Trozzi G., Istasse C., Petit A., Rasschaert P., Denesvre C., Kaufer B.B., Bertzbach L.D., Muylkens B., Coupeau D. (2022). Marek’s Disease Virus Virulence Genes Encode Circular RNAs. J. Virol..

[31] Zhang Y.S., Zhu M., Zhang X., Dai K., Liang Z., Pan J., Zhang Z.Y., Cao M.M., Xue R.Y., Cao G.L. (2022). Micropeptide vsp21 translated by Reovirus circular RNA 000048 attenuates viral replication. Int. J. Biol. Macromol..

[32] Zhang Y., Zhang X., Shen Z., Qiu Q., Tong X., Pan J., Zhu M., Hu X., Gong C. (2023). BmNPV circular RNA-encoded peptide VSP39 promotes viral replication. Int. J. Biol. Macromol..

[33] Shen Z., Li S., Liu Z., Qi Y.L., Yu W.B., Zhang X., Zhu M., Hu X.L., Gong C.L. (2024). GCRV-encoded circRNA circ_20 forms a ternary complex with BIP and PERK to delay virus replication by inhibiting the PERK-eIF2α pathway. Int. J. Biol. Macromol..

[34] Shen Z., Li S., Zhu T., Qiu Q., Gong C., Zhang X., Hu X. (2025). A novel viral circRNA-13 encoded by GCRV with delaying viral proliferation. Aquaculture.

[35] Zhu M., Dai Y., Tong X., Zhang Y., Zhou Y., Cheng J., Jiang Y., Yang R., Wang X., Cao G. (2022). Circ-Udg Derived from Cyprinid Herpesvirus 2 Promotes Viral Replication. Microbiol. Spectr..

[36] Limkul S., Phiwthong T., Wanvimonsuk S., Seabkongseng T., Aunkam P., Jaree P., Luangtrakul W., Mahanil K., Teamtisong K., Tittabutr P. (2025). Viral circular RNA-encoded protein, ceVP28, divulges an antiviral response in invertebrates. Proc. Natl. Acad. Sci. USA.

[37] Ge J., Meng Y., Guo J., Chen P., Wang J., Shi L., Wang D., Qu H., Wu P., Fan C. (2024). Human papillomavirus-encoded circular RNA circE7 promotes immune evasion in head and neck squamous cell carcinoma. Nat. Commun..

[38] Zhao J.W., Lee E.E., Kim J., Yang R., Chamseddin B., Ni C.Y., Gusho E., Xie Y., Chiang C.M., Buszczak M. (2019). Transforming activity of an oncoprotein-encoding circular RNA from human papillomavirus. Nat. Commun..

[39] Gong L.-P., Chen J.-N., Dong M., Xiao Z.-D., Feng Z.-Y., Pan Y.-H., Zhang Y., Du Y., Zhang J.-Y., Bi Y.-H. (2020). Epstein–Barr virus-derived circular RNA LMP 2A induces stemness in EBV-associated gastric cancer. EMBO Rep..

[40] Tan K.E., Ng W.L., Marinov G.K., Yu K.H.O., Tan L.P., Liau E.S., Goh S.Y., Yeo K.S., Yip K.Y., Lo K.W. (2021). Identification and characterization of a novel Epstein-Barr Virus-encoded circular RNA from Gene. Sci. Rep..

[41] Liu Q.W., Shuai M.X., Xia Y. (2019). Knockdown of EBV-encoded circRNA circRPMS1 suppresses nasopharyngeal carcinoma cell proliferation and metastasis through sponging multiple miRNAs. Cancer Manag. Res..

[42] Zhang J.Y., Du Y., Gong L.P., Shao Y.T., Pan L.J., Feng Z.Y., Pan Y.H., Huang J.T., Wen J.Y., Sun L.P. (2022). ebv-circRPMS1 promotes the progression of EBV-associated gastric carcinoma via Sam68-dependent activation of METTL3. Cancer Lett..

[43] Ungerleider N., Concha M., Lin Z., Roberts C., Wang X., Cao S.B., Baddoo M., Moss W.N., Yu Y., Seddon M. (2018). The Epstein Barr virus circRNAome. PLoS Pathog..

[44] Tagawa T., Oh D., Santos J., Dremel S., Mahesh G., Uldrick T.S., Yarchoan R., Kopardé V.N., Ziegelbauer J.M. (2021). Characterizing Expression and Regulation of Gamma-Herpesviral Circular RNAs. Front. Microbiol..

[45] Abere B., Li J.H., Zhou H.Z., Toptan T., Moore P.S., Chang Y. (2020). Kaposi’s Sarcoma-Associated Herpesvirus-Encoded circRNAs Are Expressed in Infected Tumor Tissues and Are Incorporated into Virions. mBio.

[46] Withers J.B., Li E.S., Vallery T.K., Yario T.A., Steitz J.A. (2018). Two herpesviral noncoding PAN RNAs are functionally homologous but do not associate with common chromatin loci. PLoS Pathog..

[47] Yang R., Lee E.E., Kim J., Choi J.H., Kolitz E., Chen Y.T., Crewe C., Salisbury N.J.H., Scherer P.E., Cockerell C. (2021). Characterization of ALTO-encoding circular RNAs expressed by Merkel cell polyomavirus and trichodysplasia spinulosa polyomavirus. PLoS Pathog..

[48] Mazziotta C., Cervellera C.F., Lanzillotti C., Touzé A., Gaboriaud P., Tognon M., Martini F., Rotondo J.C. (2023). MicroRNA dysregulations in Merkel cell carcinoma: Molecular mechanisms and clinical applications. J. Med. Virol..

[49] Abere B., Zhou H.Z., Li J.H., Cao S., Toptan T., Grundhoff A., Fischer N., Moore P.S., Chang Y. (2020). Merkel Cell Polyomavirus Encodes Circular RNAs (circRNAs) Enabling a Dynamic circRNA/microRNA/mRNA Regulatory Network. mBio.

[50] Zhu M., Liang Z., Pan J., Zhang X., Xue R.Y., Cao G.L., Hu X.L., Gong C.L. (2021). Hepatocellular carcinoma progression mediated by hepatitis B virus-encoded circRNA HBV_circ_1 through interaction with CDK1. Mol. Ther. Nucleic Acids.

[51] Zhu M., Liang Z., Pan J., Hu X., Zhang X., Xue R., Cao G., Gong C. (2020). HBV pgRNA can generate a circRNA with two junction sites. bioRxiv.

[52] Cao Q.M., Boonchuen P., Chen T.C., Lei S.H., Somboonwiwat K., Sarnow P. (2024). Virus- derived circular RNAs populate hepatitis C virus-infected cells. Proc. Natl. Acad. Sci. USA.

[53] Wen Y.H., Zhao H.L., Wu S.Y., Jiang J.X., Gao Y., Wang Z.F., Liu X.Y., Yu F., Ou T., Zhao A.Z. (2025). CircSARS-CV2-N1368 from SARS-CoV-2 impairs endothelial cell function through the upregulation of ATF7 to activate TLR4/NF-κB/ROS signaling. Acta Pharmacol. Sin..

[54] Zhu C., Wang J., Du Y., Li C., Hao M., Zhang Y., Zhang X., Guan Y., Zheng F., Zhang Y. (2023). Influenza A virus H1N1-derived circNP37 positively regulates viral replication by sponging host miR-361-5p. bioRxiv.

[55] Diener T.O. (2003). Discovering viroids__A personal perspective. Nat. Rev. Microbiol..

[56] Diener T.O. (1972). Viroids. Adv. Virus Res..

[57] Pan J., Zhang X., Zhang Y.S., Yan B.Y., Dai K., Zhu M., Liang Z., Dai Y.P., Zhang M.T., Zhang Z.Y. (2021). Grass carp reovirus encoding circular RNAs with antiviral activity. Aquaculture.

[58] Sekiba K., Otsuka M., Ohno M., Kishikawa T., Yamagami M., Suzuki T., Ishibashi R., Seimiya T., Tanaka E., Koike K. (2018). DHX9 regulates production of hepatitis B virus-derived circular RNA and viral protein levels. Oncotarget.

[59] Cai Z., Lu C., He J., Liu L., Zou Y., Zhang Z., Zhu Z., Ge X., Wu A., Jiang T. (2021). Identification and characterization of circRNAs encoded by MERS-CoV, SARS-CoV-1 and SARS-CoV-2. Brief bioinform.

[60] Liu B., Yuan R., Liang Z., Zhang T.T., Zhu M., Zhang X., Geng W., Fang P., Jiang M.S., Wang Z.Y. (2019). Comprehensive analysis of circRNA expression pattern and circRNA-mRNA-miRNA network in kidney (CIK) cells after grass carp reovirus (GCRV) infection. Aquaculture.

[61] Su H., Zheng W., Pan J., Lv X., Xin S., Xu T. (2021). Circular RNA circSamd4a regulates antiviral immunity in teleost fish by upregulating STING through sponging miR-29a-3p. J. Immunol..

[62] Lou Y.Y., Wang Q.D., Lu Y.T., Tu M.Y., Xu X., Xia Y., Peng Y., Lai M.M., Zheng X.Q. (2019). Differential circRNA expression profiles in latent human cytomegalovirus infection and validation using clinical samples. Physiol. Genom..

[63] Lu S., Zhu N., Guo W.W., Wang X., Li K.J., Yan J., Jiang C.P., Han S.Y., Xiang H.M., Wu X.H. (2020). RNA-Seq Revealed a Circular RNA-microRNA-mRNA Regulatory Network in Hantaan Virus Infection. Front. Cell. Infect. Microbiol..

[64] Li X., Liu C.X., Xue W., Zhang Y., Jiang S., Yin Q.F., Wei J., Yao R.W., Yang L., Chen L.L. (2017). Coordinated circRNA Biogenesis and Function with NF90/NF110 in Viral Infection. Mol. Cell.

[65] Liu C.X., Guo S.K., Nan F., Xu Y.F., Yang L., Chen L.L. (2022). RNA circles with minimized immunogenicity as potent PKR inhibitors. Mol. Cell.

[66] Liu C.X., Li X., Nan F., Jiang S., Gao X., Guo S.K., Xue W., Cui Y.G., Dong K.G., Ding H.H. (2019). Structure and Degradation of Circular RNAs Regulate PKR Activation in Innate Immunity. Cell.

[67] Nallagatla S.R., Toroney R., Bevilacqua P.C. (2011). Regulation of innate immunity through RNA structure and the protein kinase PKR. Curr. Opin. Struct. Biol..

[68] Su Q., Wang S., Baltzis D., Qu L.K., Wong A.H., Koromilas A.E. (2006). Tyrosine phosphorylation acts as a molecular switch to full-scale activation of the eIF2alpha RNA-dependent protein kinase. Proc. Natl. Acad. Sci. USA.

[69] Balachandran S., Roberts P.C., Brown L.E., Truong H., Pattnaik A.K., Archer D.R., Barber G.N. (2000). Essential role for the dsRNA-dependent protein kinase PKR in innate immunity to viral infection. Immunity.

[70] Gil J., Rullas J., García M.A., Alcamí J., Esteban M. (2001). The catalytic activity of dsRNA-dependent protein kinase, PKR, is required for NF-kappaB activation. Oncogene.

[71] Zhang L., Alter H.J., Wang H., Jia S., Wang E., Marincola F.M., Shih J.W., Wang R.Y. (2013). The modulation of hepatitis C virus 1a replication by PKR is dependent on NF-kB mediated interferon beta response in Huh7.5.1 cells. Virology.

[72] Lee S.B., Esteban M. (1994). The interferon-induced double-stranded RNA-activated protein kinase induces apoptosis. Virology.

[73] Kibler K.V., Shors T., Perkins K.B., Zeman C.C., Banaszak M.P., Biesterfeldt J., Langland J.O., Jacobs B.L. (1997). Double-stranded RNA is a trigger for apoptosis in vaccinia virus-infected cells. J. Virol..

[74] Chen Y.G., Kim M.V., Chen X., Batista P.J., Aoyama S., Wilusz J.E., Iwasaki A., Chang H.Y. (2017). Sensing Self and Foreign Circular RNAs by Intron Identity. Mol. Cell.

[75] Chen Y.G., Chen R., Ahmad S., Verma R., Kasturi S.P., Amaya L., Broughton J.P., Kim J., Cadena C., Pulendran B. (2019). N6-Methyladenosine Modification Controls Circular RNA Immunity. Mol. Cell.

[76] Onomoto K., Onoguchi K., Yoneyama M. (2021). Regulation of RIG-I-like receptor-mediated signaling: Interaction between host and viral factors. Cell Mol. Immunol..

[77] Lv X., Zheng W., Geng S., Cui Y., Tao Y., Xu T. (2023). circCBL and its host gene CBL collaboratively enhance the antiviral immunity and antibacterial immunity by targeting MITA in fish. J. Virol..

[78] Qiu H., Yang B., Chen Y., Zhu Q., Wen F., Peng M., Wang G., Guo G., Chen B., Maarouf M. (2023). Influenza A Virus-Induced circRNA circMerTK Negatively Regulates Innate Antiviral Responses. Microbiol. Spectr..

[79] Zhang L.D., Wang Z.C. (2020). Circular RNA hsa_circ_0004812 impairs IFN-induced immune response by sponging miR-1287-5p to regulate FSTL1 in chronic hepatitis B. Virol. J..

[80] Jiang W.X., Wang L.L., Zhang Y.J., Li H.L. (2020). Circ-ATP5H Induces Hepatitis B Virus Replication and Expression by Regulating miR-138-5p/*TNFAIP3* Axis. Cancer Manag. Res..

[81] Li H., Du L., Li J., Huang Y., Lu C., Deng T., Yan Y., Jin Y., Wu W., Gu J. (2024). A previously unidentified circRNA inhibits virus replication by regulating the miR-24-3p/KEAP1 axis. PLoS Pathog..

[82] Zheng W.W., Zhu X.X., Zhu T.T., Luo Q., Zhao Y., Xu T.J. (2025). A Novel Protein NLRP12-119aa that Prevents Rhabdovirus Replication by Disrupting the RNP Complex Formation. Adv. Sci..

[83] Wang L., Zheng G., Yuan Y., Wang Z., Wang Q., Sun M., Wu J., Liu C., Liu Y., Zhang B. (2024). circRUNX2.2, highly expressed in Marek’s disease tumor tissues, functions in cis to regulate parental gene RUNX2 expression. Poult. Sci..

[84] Kang L., Xie H., Ye H., Jeyarajan A.J., Warner C.A., Huang Y., Shi Y., Li Y., Yang C., Xu M. (2023). Hsa_circ_0007321 regulates Zika virus replication through miR-492/NFKBID/NF-κB signaling pathway. J. Virol..

[85] Zhang X., Chu H., Chik K.K.H., Wen L., Shuai H.P., Yang D., Wang Y.X., Hou Y.X., Yuen T.T.T., Cai J.P. (2022). hnRNP C modulates MERS-CoV and SARS-CoV-2 replication by governing the expression of a subset of circRNAs and cognitive mRNAs. Emerg. Microbes Infect..

[86] Shi N., Zhang S., Guo Y., Yu X., Zhao W., Zhang M., Guan Z., Duan M. (2021). CircRNA_0050463 promotes influenza A virus replication by sponging miR-33b-5p to regulate EEF1A1. Vet. Microbiol..

[87] Du L., Wang X., Liu J., Li J., Wang S., Lei J., Zhou J., Gu J. (2021). A Previously Undiscovered Circular RNA, circTNFAIP3, and Its Role in Coronavirus Replication. mBio.

[88] Kristensen L.S., Jakobsen T., Hager H., Kjems J. (2022). The emerging roles of circRNAs in cancer and oncology. Nat. Rev. Clin. Oncol..

[89] Li Y., Zeng X., He J., Gui Y., Zhao S., Chen H., Sun Q., Jia N., Yuan H. (2018). Circular RNA as a biomarker for cancer: A systematic meta-analysis. Oncol. Lett..

[90] Su Y., Zhong G., Jiang N., Huang M., Lin T. (2018). Circular RNA, a novel marker for cancer determination (Review). Int. J. Mol. Med..

[91] Yu J., Ding W.B., Wang M.C., Guo X.G., Xu J., Xu Q.G., Yang Y., Sun S.H., Liu J.F., Qin L.X. (2020). Plasma circular RNA panel to diagnose hepatitis B virus-related hepatocellular carcinoma: A large-scale, multicenter study. Int. J. Cancer.

[92] Memczak S., Papavasileiou P., Peters O., Rajewsky N. (2015). Identification and Characterization of Circular RNAs As a New Class of Putative Biomarkers in Human Blood. PLoS ONE.

[93] Yang R., Wang R.C. (2021). Research techniques made simple: Studying circular RNA in skin diseases. J. Investig. Dermatol..

[94] Shuai M., Huang L. (2020). High Expression of hsa_circRNA_001387 in Nasopharyngeal Carcinoma and the Effect on Efficacy of Radiotherapy. Onco Targets Ther..

[95] Hong X., Liu N., Liang Y., He Q., Yang X., Lei Y., Zhang P., Zhao Y., He S., Wang Y. (2020). Circular RNA CRIM1 functions as a ceRNA to promote nasopharyngeal carcinoma metastasis and docetaxel chemoresistance through upregulating FOXQ1. Mol Cancer.

[96] Xie Y., Shao Y., Sun W., Ye G., Zhang X., Xiao B., Guo J. (2018). Downregulated expression of hsa_circ_0074362 in gastric cancer and its potential diagnostic values. Biomark. Med..

[97] Li P., Chen S., Chen H., Mo X., Li T., Shao Y., Xiao B., Guo J. (2015). Using circular RNA as a novel type of biomarker in the screening of gastric cancer. Clin. Chim. Acta.

[98] Sun H., Tang W., Rong D., Jin H., Fu K., Zhang W., Liu Z., Cao H., Cao X. (2018). Hsa_circ_0000520, a potential new circular RNA biomarker, is involved in gastric carcinoma. Cancer Biomark..

[99] Li T., Shao Y., Fu L., Xie Y., Zhu L., Sun W., Yu R., Xiao B., Guo J. (2018). Plasma circular RNA profiling of patients with gastric cancer and their droplet digital RT-PCR detection. J. Mol. Med..

[100] Zhu K., Zhan H., Peng Y., Yang L., Gao Q., Jia H., Dai Z., Tang Z., Fan J., Zhou J. (2020). Plasma hsa_circ_0027089 is a diagnostic biomarker for hepatitis B virus-related hepatocellular carcinoma. Carcinogenesis.

[101] Ishaq Y., Rauff B., Alzahrani B., Javed H., Ikram A. (2024). Identification of Serum-Derived CricRNA Diagnostic Panel and Revealing Their Regulatory Mechanisms in HCV-HCC: A Cross-Sectional Study. Health Sci. Rep..

[102] Zhao S.Y., Wang J., Ouyang S.B., Huang Z.K., Liao L. (2018). Salivary Circular RNAs Hsa_Circ_0001874 and Hsa_Circ_0001971 as Novel Biomarkers for the Diagnosis of Oral Squamous Cell Carcinoma. Cell Physiol. Biochem..

[103] Zhang X., Yang S., Chen W., Dong X., Zhang R., Ye H., Mei X., Liu H., Fang Y., He C. (2021). Circular RNA circYPEL2: A Novel Biomarker in Cervical Cancer. Genes.

[104] Li N., Liu J., Deng X. (2021). Identification of a novel circRNA, hsa_circ_0065898, that regulates tumor growth in cervical squamous cell carcinoma. Transl. Cancer Res..

[105] Zhao T., Zheng Y., Hao D., Jin X., Luo Q., Guo Y., Li D., Xi W., Xu Y., Chen Y. (2019). Blood circRNAs as biomarkers for the diagnosis of community-acquired pneumonia. J. Cell Biochem..

[106] He J., Ming Y., MinLi Y., Han Z., Jiang J., Zhou J., Dai B., Lv Y., He M.L., Fang M. (2019). hsa_circ_0006459 and hsa_circ_0015962 affect prognosis of Dengue fever. Sci. Rep..

[107] Yang D., Sun K., Huang F., Fan H., Shi T., Chen X., Lu G. (2022). Whole blood circular RNA hsa_circ_0002171 serves as a potential diagnostic biomarker for human adenovirus pneumonia in children. Braz. J. Med. Biol. Res..

[108] Wang M., Gu B., Yao G., Li P., Wang K. (2020). Circular RNA Expression Profiles and the Pro-tumorigenic Function of CircRNA_10156 in Hepatitis B Virus-Related Liver Cancer. Int. J. Med. Sci..

[109] Zhao J., Zhang T., Wu P., Qiu J., Wu K., Shi L., Zhu Q., Zhou J. (2024). Circrna-0015004 act as a ceRNA to promote RCC2 expression in hepatocellular carcinoma. Sci. Rep..

[110] Xu H., Zheng Y., Wu J., Zhang R., Zhao Q., Chen S., Peng W., Cai D., Gao Y., Chen X. (2024). circSORBS1 inhibits lung cancer progression by sponging miR-6779-5p and directly binding RUFY3 mRNA. J. Transl. Med..

[111] Huang K., Huang D., Li Q., Zeng J., Qin T., Zhong J., Zhong Z., Lu S. (2024). CircSorbs1 regulates myocardial regeneration and reduces cancer therapy-related cardiovascular toxicity through the Mir-99/GATA4 pathway. Discov. Oncol..

[112] Tagawa T., Gao S., Koparde V.N., Gonzalez M., Spouge J.L., Serquiña A.P., Lurain K., Ramaswami R., Uldrick T.S., Yarchoan R. (2018). Discovery of Kaposi’s sarcoma herpesvirus-encoded circular RNAs and a human antiviral circular RNA. Proc. Natl. Acad. Sci. USA.

[113] Tagawa T., Oh D., Dremel S., Mahesh G., Koparde V.N., Duncan G., Andresson T., Ziegelbauer J.M. (2023). A virus-induced circular RNA maintains latent infection of Kaposi’s sarcoma herpesvirus. Proc. Natl. Acad. Sci. USA.

[114] Zhang X., Chu H., Wen L., Shuai H., Yang D., Wang Y., Hou Y., Zhu Z., Yuan S., Yin F.F. (2020). Competing endogenous RNA network profiling reveals novel host dependency factors required for MERS-CoV propagation. Emerg. Microbes Infect..

[115] Pfafenrot C., Schneider T., Müller C., Hung L.H., Schreiner S., Ziebuhr J., Bindereif A. (2021). Inhibition of SARS-CoV-2 coronavirus proliferation by designer antisense-circRNAs. Nucleic Acids Res..

[116] Wang Z.Y., Guo Z.D., Li J.M., Zhao Z.Z., Fu Y.Y., Zhang C.M., Zhang Y., Liu L.N., Qian J., Liu L.N. (2017). Genome-Wide Search for Competing Endogenous RNAs Responsible for the Effects Induced by Ebola Virus Replication and Transcription Using a trVLP System. Front. Cell Infect. Microbiol..

[117] Liu G., Zhu D., Feng K., Peng H., Yang S., Huang L., Li P. (2025). The neurological damage caused by enterovirus 71 infection is associated with hsa_circ_0069335/miR-29b/PMP22 pathway. J. Virol..

[118] Li S., Li X., Xue W., Zhang L., Yang L.Z., Cao S.M., Lei Y.N., Liu C.X., Guo S.K., Shan L. (2021). Screening for functional circular RNAs using the CRISPR-Cas13 system. Nat. Methods.

[119] Zhan Y., Cao C., Li A., Mei H., Liu Y. (2023). Enhanced RNA knockdown efficiency with engineered fusion guide RNAs that function with both CRISPR-CasRx and hammerhead ribozyme. Genome Biol..

[120] Jost I., Shalamova L.A., Gerresheim G.K., Niepmann M., Bindereif A., Rossbach O. (2018). Functional sequestration of microRNA-122 from Hepatitis C Virus by circular RNA sponges. RNA Biol..

[121] Plotkin S.A., Plotkin S.L. (2011). The development of vaccines: How the past led to the future. Nat. Rev. Microbiol..

[122] Wesselhoeft R.A., Kowalski P.S., Parker-Hale F.C., Huang Y., Bisaria N., Anderson D.G. (2019). RNA Circularization Diminishes Immunogenicity and Can Extend Translation Duration In Vivo. Mol. Cell.

[123] Liu X., Zhang Y., Zhou S., Dain L., Mei L., Zhu G. (2022). Circular RNA: An emerging frontier in RNA therapeutic targets, RNA therapeutics, and mRNA vaccines. J. Control Release.

[124] Wesselhoeft R.A., Kowalski P.S., Anderson D.G. (2018). Engineering circular RNA for potent and stable translation in eukaryotic cells. Nat. Commun..

[125] Qu L., Yi Z., Shen Y., Lin L., Chen F., Xu Y., Wu Z., Tang H., Zhang X., Tian F. (2022). Circular RNA vaccines against SARS-CoV-2 and emerging variants. Cell.

[126] Zhou J., Ye T., Yang Y., Li E., Zhang K., Wang Y., Chen S., Hu J., Zhang K., Liu F. (2024). Circular RNA vaccines against monkeypox virus provide potent protection against vaccinia virus infection in mice. Mol. Ther..

[127] Wan J., Wang Z., Wang L., Wu L., Zhang C., Zhou M., Fu Z.F., Zhao L. (2024). Circular RNA vaccines with long-term lymph node-targeting delivery stability after lyophilization induce potent and persistent immune responses. mBio.

[128] Yue X., Zhong C., Cao R., Liu S., Qin Z., Liu L., Zhai Y., Luo W., Lian Y., Zhang M. (2024). CircRNA based multivalent neuraminidase vaccine induces broad protection against influenza viruses in mice. NPJ Vaccines.

[129] Amaya L., Grigoryan L., Li Z., Lee A., Wender P.A., Pulendran B., Chang H.Y. (2023). Circular RNA vaccine induces potent T cell responses. Proc. Natl. Acad. Sci. USA.

[130] Wan J., Wang C., Wang Z., Wang L., Wang H., Zhou M., Fu Z.F., Zhao L. (2024). CXCL13 promotes broad immune responses induced by circular RNA vaccines. Proc. Natl. Acad. Sci. USA.

[131] Bu T., Yang Z.Y., Zhao J., Gao Y.M., Li F.X., Yang R. (2025). Expanding the Potential of Circular RNA (CircRNA) Vaccines: A Promising Therapeutic Approach. Int. J. Mol. Sci..

[132] Xie J., Ye F., Deng X., Tang Y., Liang J.Y., Huang X., Sun Y., Tang H., Lei J., Zheng S. (2023). Circular RNA: A promising new star of vaccine. J. Transl. Int. Med..

[133] Liang R., He Z., Zhao K.T., Zhu H., Hu J., Liu G., Gao Q., Liu M., Zhang R., Qiu J.L. (2024). Prime editing using CRISPR-Cas12a and circular RNAs in human cells. Nat. Biotechnol..

[134] Su C.I., Chuang Z.S., Shie C.T., Wang H.I., Kao Y.T., Yu C.Y. (2024). A cis-acting ligase ribozyme generates circular RNA in vitro for ectopic protein functioning. Nat. Commun..

[135] Attwaters M. (2022). In vivo RNA base editing with circular RNAs. Nat. Rev. Genet..

[136] Katrekar D., Yen J., Xiang Y., Saha A., Meluzzi D., Savva Y., Mali P. (2022). Efficient in vitro and in vivo RNA editing via recruitment of endogenous ADARs using circular guide RNAs. Nat. Biotechnol..

[137] Yi Z., Qu L., Tang H., Liu Z., Liu Y., Tian F., Wang C., Zhang X., Feng Z., Yu Y. (2022). Engineered circular ADAR-recruiting RNAs increase the efficiency and fidelity of RNA editing in vitro and in vivo. Nat. Biotechnol..

[138] Zhang X., Li M., Chen K., Liu Y., Liu J., Wang J., Huang H., Zhang Y., Huang T., Ma S. (2025). Engineered circular guide RNAs enhance miniature CRISPR/Cas12f-based gene activation and adenine base editing. Nat. Commun..

[139] Liang R., Wang S., Cai Y., Li Z., Li K.M., Wei J., Sun C., Zhu H., Chen K., Gao C. (2025). Circular RNA-mediated inverse prime editing in human cells. Nat. Commun..

[140] Chen C.Y., Sarnow P. (1995). Initiation of protein synthesis by the eukaryotic translational apparatus on circular RNAs. Science.

[141] Pamudurti N.R., Bartok O., Jens M., Ashwal-Fluss R., Stottmeister C., Ruhe L., Hanan M., Wyler E., Perez-Hernandez D., Ramberger E. (2017). Translation of CircRNAs. Mol. Cell.

[142] Hwang H.J., Kim Y.K. (2024). Molecular mechanisms of circular RNA translation. Exp. Mol. Med..

[143] Abe N., Hiroshima M., Maruyama H., Nakashima Y., Nakano Y., Matsuda A., Sako Y., Ito Y., Abe H. (2013). Rolling circle amplification in a prokaryotic translation system using small circular RNA. Angew. Chem. Int. Ed. Engl..

[144] Abe N., Matsumoto K., Nishihara M., Nakano Y., Shibata A., Maruyama H., Shuto S., Matsuda A., Yoshida M., Ito Y. (2015). Rolling Circle Translation of Circular RNA in Living Human Cells. Sci. Rep..

[145] Nakamoto K., Abe N., Tsuji G., Kimura Y., Tomoike F., Shimizu Y., Abe H. (2020). Chemically synthesized circular RNAs with phosphoramidate linkages enable rolling circle translation. Chem. Commun..

[146] Lin H.H., Chang C.Y., Huang Y.R., Shen C.H., Wu Y.C., Chang K.L., Lee Y.C., Lin Y.C., Ting W.C., Chien H.J. (2023). Exon Junction Complex Mediates the Cap-Independent Translation of Circular RNA. Mol. Cancer Res..

[147] Hanson P.J., Zhang H.M., Hemida M.G., Ye X., Qiu Y., Yang D. (2012). IRES-Dependent Translational Control during Virus-Induced Endoplasmic Reticulum Stress and Apoptosis. Front. Microbiol..

[148] Komastu T., Ireland D.D., Reiss C.S. (1998). IL-12 and viral infections. Cytokine Growth Factor. Rev..

[149] Li Y., Gan J., Lei J., Qi S., Yu X., Zhang W., Feng Y., Zhang Y., Cheng M., Ma L. (2025). Catalytic Hybrid Lipid Nanoparticles Potentiate Circle RNA-Based Cytokine Immunotherapy. ACS Nano.

[150] Woodruff R., Parekh F., Lamb K., Mekkaoui L., Allen C., Smetanova K., Huang J., Williams A., Toledo G.S., Lilova K. (2023). Large-scale manufacturing of base-edited chimeric antigen receptor T cells. Mol. Ther. Methods Clin. Dev..

[151] Ma Y., Wang T., Zhang X., Wang P., Long F. (2024). The role of circular RNAs in regulating resistance to cancer immunotherapy: Mechanisms and implications. Cell Death Dis..

[152] Huang S., Xu J., Baran N., Ma W. (2024). Advancing the next generation of cancer treatment with circular RNAs in CAR-T cell therapy. Biomed. Pharmacother..

[153] Shen L., Yang J., Zuo C., Xu J., Ma L., He Q., Zhou X., Ding X., Wei L., Jiang S. (2024). Circular mRNA-based TCR-T offers a safe and effective therapeutic strategy for treatment of cytomegalovirus infection. Mol. Ther..

